# Sex-specific associations of adiposity with cardiometabolic traits in the UK: A multi–life stage cohort study with repeat metabolomics

**DOI:** 10.1371/journal.pmed.1003636

**Published:** 2022-01-06

**Authors:** Linda M. O’Keeffe, Joshua A. Bell, Kate N. O’Neill, Matthew A. Lee, Mark Woodward, Sanne A. E. Peters, George Davey Smith, Patricia M. Kearney

**Affiliations:** 1 School of Public Health, University College Cork, Cork, Ireland; 2 MRC Integrative Epidemiology Unit at the University of Bristol, Bristol, United Kingdom; 3 Population Health Sciences, Bristol Medical School, University of Bristol, Bristol, United Kingdom; 4 The George Institute for Global Health, School of Public Health, Imperial College, London, United Kingdom; 5 The George Institute for Global Health, University of New South Wales, Sydney, Australia; 6 Julius Centre for Health Sciences and Primary Care, University Medical Centre Utrecht, Utrecht University, Utrecht, the Netherlands; Shanghai Jiao Tong University Affiliated Sixth People’s Hospital, CHINA

## Abstract

**Background:**

Sex differences in cardiometabolic disease risk are commonly observed across the life course but are poorly understood and may be due to different associations of adiposity with cardiometabolic risk in females and males. We examined whether adiposity is differently associated with cardiometabolic trait levels in females and males at 3 different life stages.

**Methods and findings:**

Data were from 2 generations (offspring, Generation 1 [G1] born in 1991/1992 and their parents, Generation 0 [G0]) of a United Kingdom population-based birth cohort study, the Avon Longitudinal Study of Parents and Children (ALSPAC). Follow-up continues on the cohort; data up to 25 y after recruitment to the study are included in this analysis. Body mass index (BMI) and total fat mass from dual-energy X-ray absorptiometry (DXA) were measured at mean age 9 y, 15 y, and 18 y in G1. Waist circumference was measured at 9 y and 15 y in G1. Concentrations of 148 cardiometabolic traits quantified using nuclear magnetic resonance spectroscopy were measured at 15 y, 18 y, and 25 y in G1. In G0, all 3 adiposity measures and the same 148 traits were available at 50 y. Using linear regression models, sex-specific associations of adiposity measures at each time point (9 y, 15 y, and 18 y) with cardiometabolic traits 3 to 6 y later were examined in G1. In G0, sex-specific associations of adiposity measures and cardiometabolic traits were examined cross-sectionally at 50 y. A total of 3,081 G1 and 4,887 G0 participants contributed to analyses. BMI was more strongly associated with key atherogenic traits in males compared with females at younger ages (15 y to 25 y), and associations were more similar between the sexes or stronger in females at 50 y, particularly for apolipoprotein B–containing lipoprotein particles and lipid concentrations. For example, a 1 standard deviation (SD) (3.8 kg/m^2^) higher BMI at 18 y was associated with 0.36 SD (95% confidence interval [CI] = 0.20, 0.52) higher concentrations of extremely large very-low-density lipoprotein (VLDL) particles at 25 y in males compared with 0.15 SD (95% CI = 0.09, 0.21) in females, *P* value for sex difference = 0.02. By contrast, at 50 y, a 1 SD (4.8 kg/m^2^) higher BMI was associated with 0.33 SD (95% CI = 0.25, 0.42) and 0.30 SD (95% CI = 0.26, 0.33) higher concentrations of extremely large VLDL particles in males and females, respectively, *P* value for sex difference = 0.42. Sex-specific associations of DXA-measured fat mass and waist circumference with cardiometabolic traits were similar to findings for BMI and cardiometabolic traits at each age. The main limitation of this work is its observational nature, and replication in independent cohorts using methods that can infer causality is required.

**Conclusions:**

The results of this study suggest that associations of adiposity with adverse cardiometabolic risk begin earlier in the life course among males compared with females and are stronger until midlife, particularly for key atherogenic lipids. Adolescent and young adult males may therefore be high priority targets for obesity prevention efforts.

## Abstract 2

Linda O’Keeffe and colleagues investigate sex-specific associations between cardiometabolomic traits and BMI, fat mass, and waist circumference across childhood, adolescence, early adulthood, and mid life in two generations of a UK population-based birth cohort.

## Author summary

**Why was this study done?:**

- Females and males have different cardiovascular disease (CVD) risk across the life course, but the reasons for this are unclear.
- Different associations of adiposity with CVD risk in females and males at different life stages may play a role, but this remains poorly understood.

**What did the researchers do and find?:**

- We used data from 2 generations of a UK cohort study which included 3,081 offspring born in 1991/1992 and 4,887 parents also recruited in 1991/1992 and followed up for 25 y.
- Using different measures of adiposity (body mass index [BMI], total fat mass, and waist circumference), we examined the association between adiposity and 148 traits related to CVD in females and males separately at 3 life stages: adolescence, early adulthood, and midlife.
- Our findings showed that adiposity, regardless of the measure used, was more strongly associated with key traits related to CVD in males at younger ages (15 y to 25 y).
- By contrast, associations of adiposity with these traits were more similar between the sexes or stronger in females at 50 y.

**What do these findings mean?:**

- Findings suggest that associations of adiposity with cardiometabolic traits among females and males are likely to differ across different life stages.
- These findings may have implications for adiposity prevention efforts in females and males across the life course, suggesting that adolescent and young adult males may be a priority group for adiposity prevention.
- Replication in other cohorts using methods that can infer causality is required.

## Introduction

Cardiometabolic diseases including type 2 diabetes mellitus (T2DM) and cardiovascular disease (CVD) are a leading cause of death worldwide [1]. Females and males do not experience this risk equally; for instance, males develop higher systolic blood pressure (SBP) during adolescence, and this sex difference widens in early adulthood and persists throughout much of the life course [2–4]. Furthermore, age-adjusted CVD risk is higher in males compared with females throughout adulthood, and, although this sex difference attenuates with age, female and male CVD risk only become similar from approximately the seventh or eighth decade onwards [5]. The underlying aetiology of this sex difference remains poorly understood, however, and opportunities to develop and deliver more effective sex-specific CVD prevention across the life course are underexplored.

Emerging evidence suggests that the cardiometabolic consequences of adiposity may differ in females and males [6,7], contributing to sex differences in cardiometabolic disease risk. To date, several studies have examined sex-specific associations of adiposity with clinical end points such as coronary heart disease (CHD) in adults [8–16], although results have been conflicting and based mostly on body mass index (BMI). By contrast, few studies have examined sex-specific associations of adiposity with cardiometabolic trait measures from metabolomics platforms that provide more granular insight into CVD aetiology [7]. Moreover, the cardiometabolic consequences of adiposity among females and males across different life stages have not been investigated [8–16], despite potentially important implications for the timing of disease prevention across the life course.

Using data from 2 generations of the Avon Longitudinal Study of Parents and Children (ALSPAC) birth cohort, we compared the sex-specific associations of BMI, dual-energy X-ray absorptiometry (DXA)-determined total fat mass, and waist circumference with cardiometabolic traits from targeted metabolomics measured 3 times among offspring (Generation 1 [G1] cohort in mid adolescence, late adolescence, and early adulthood) and once in midlife among their parents (Generation 0 [G0] cohort).

## Methods

### Study population

Data were from participants of ALSPAC, a population-based birth cohort study in which 14,541 pregnant women expected to deliver between April 1, 1991 and December 31, 1992 were recruited from the former county of Avon in South West England. Offspring (G1 cohort) alive at 1 y (*n* = 13,988) have been followed up with multiple assessments with an additional 913 children enrolled over the course of the study (total = 14,901) [17,18]. Parents (G0 cohort) of G1 offspring participants have also been followed with multiple assessments [19]. Parental data used here were primarily from mothers who attended a clinic assessment between December 2008 and July 2011 and from fathers who attended a clinic assessment between September 2011 and February 2013. Study data among G1 cohort participants after age 22 y were collected and managed using REDCap electronic data capture tools hosted at University of Bristol [20,21].

Ethical approval was obtained from the ALSPAC Law and Ethics and Local Research Ethics Committees. The study website contains details of all the data that is available through a fully searchable data dictionary and variable search tool (http://www.bristol.ac.uk/alspac/researchers/our-data).

### Prespecified study protocol

A general study protocol was written in October 2018 for an ALSPAC data proposal prior to analyses (S1 Appendix) as part of a wider investigation into sex differences in CVD risk across the life course. No further changes to analyses were performed following peer review.

### Data

#### Assessment of adiposity

In the G1 cohort, BMI and DXA-determined total fat mass were measured at mean age 9 y, 15 y, and 18 y, with the additional measurement of waist circumference at 9 y and 15 y. In the G0 cohort, all 3 measures were available at 50 y. Weight was recorded to the nearest 0.1 kg using a Tanita scale. Height was measured in light clothing without shoes to the nearest 0.1 cm using a Harpenden stadiometer. BMI was calculated as weight (in kilogrammes) divided by the square of height (in metres). Total body fat mass (in kg, less head) was derived from whole body DXA scans using a GE Lunar Prodigy (Madison, Wisconsin, United States of America) narrow fan beam densitometer. Waist circumference was measured using a flexible tape to measure circumference to the nearest 1 mm at the midpoint between the lower ribs and the pelvic bone.

#### Assessment of cardiometabolic traits

In the G1 cohort, blood samples were drawn in clinics at ages 15 y, 18 y, and 25 y. Bloods were taken after a minimum of a 6-hour fast. Proton nuclear magnetic resonance (^1^H-NMR) spectroscopy from a targeted metabolomics platform [22] was performed on EDTA plasma samples from each of these 3 occasions to quantify 148 metabolite concentrations including cholesterol, triglyceride, and other lipid content in lipoprotein subclass particles, apolipoproteins, fatty acids, amino acids, and inflammatory glycoprotein acetyls.

In the G0 cohort, blood samples were drawn in clinics at mean 48 y in mothers and mean 53 y in fathers (combined mean [standard deviation, SD] age = 50 y [5 y]). Bloods were taken after a minimum of a 6-hour fast. The ^1^H-NMR metabolomics platform used in the G1 cohort was performed on serum samples taken on the G0 cohort to quantify the same 148 metabolite concentrations. In mothers only, additional blood samples were available from visits when mothers were aged 51 y, from which additional measures of the same 148 cardiometabolic concentrations were quantified. In this study, if mothers had missing data on all traits on the first measurement occasion (age 48 y) but had data on at least 1 trait on the second measurement occasion (51 y), the trait from the second occasion was used to replace missing values on the first occasion. The number of mothers with these replacements ranged from 215 to 223 across traits. Further information on the measurement of NMR traits is included in S2 Appendix and is also described elsewhere [23–25].

#### Assessment of confounders

Prior to analysis, we used directed acyclic graphs (DAGs) to identify potential confounders for each analysis performed. In the G1 cohort analyses, we adjusted for age at clinic completion, ethnicity, education of the child’s mother and father, maternal smoking during pregnancy, birth weight, gestational age, maternal age, parental household social class measured close to the time of offspring birth, and height and height^2^ at the time of exposure measurement. In analyses which included trait concentrations at 18 y and 25 y as outcomes, we also additionally adjusted for G1 offspring smoking. In the G0 cohort analyses, we adjusted for age at clinic completion, ethnicity, own education, smoking during G1 cohort pregnancy, own social class measured during the G1 child’s pregnancy, and height and height^2^ at the time of exposure measurement.

A questionnaire at 32 weeks gestation asked mothers to report their ethnicity; categories included White, Black Caribbean, Black African, Other black, Indian, Pakistani, Bangladeshi, Chinese, and Other. For the purposes of this study, ethnicity was categorised as White and UK ethnic minorities, which included all other ethnicities. A questionnaire at 32 weeks gestation asked mothers to report educational attainment for themselves and their partners separately, which was categorised as below Ordinary Level (O level; exams taken in different subjects usually at age 15 to 16 at the completion of legally required school attendance, equivalent to today’s UK General Certificate of Secondary Education), O level only, Advanced Level (A level; exams taken in different subjects usually at age 18), or university degree or above. Smoking (categorised as yes/no here) in the first trimester of pregnancy was self-reported by mothers on their own behalf and on behalf of their partners (whether they currently smoke) at 18 weeks gestation. Birth weight was extracted from medical records. Gestational age at birth was estimated from clinical records. Maternal age was reported in the mother’s antenatal questionnaires. Social class was measured using data on job title and details of occupation collected about the mother and her partner from the mother’s questionnaire at 32 weeks gestation. Social class was derived using the standard occupational classification (SOC) codes developed by the UK Office of Population Census and Surveys and classified as I professional, II managerial and technical, IIINM nonmanual, IIIM manual, and IV and V part skilled occupations and unskilled occupations. Household social class was measured as the highest of the mother’s or her partner’s occupational social class. Height was measured in light clothing without shoes to the nearest 0.1 cm using a Harpenden stadiometer. Smoking was measured via questionnaire (categorised here as yes/no) at 15 y and 18 y in the G1 cohort.

### Participants eligible for analysis

**Fig 1:**
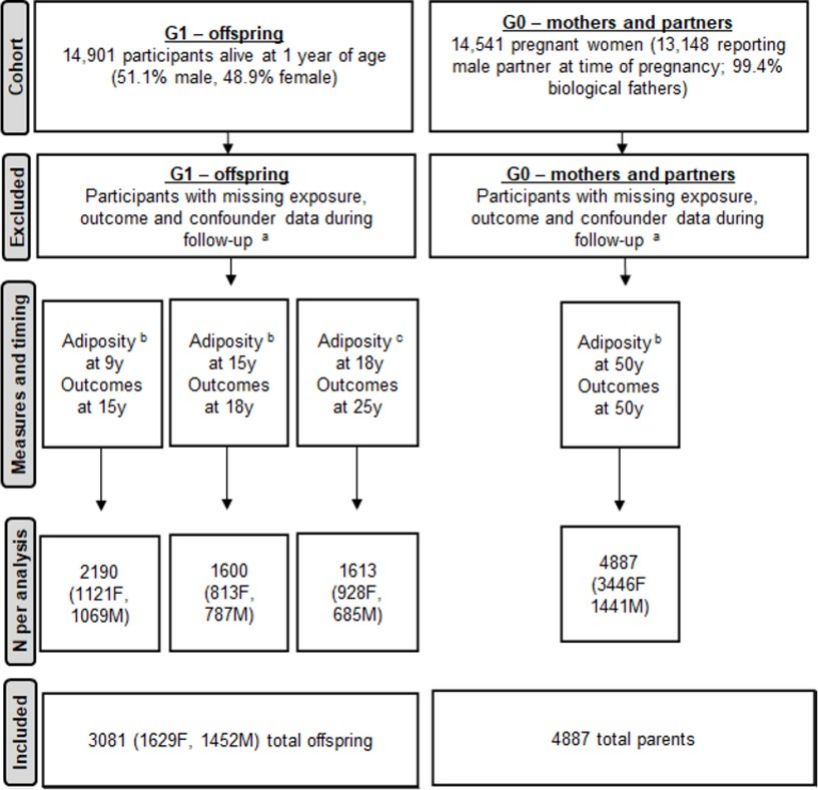
Flow diagram of study design. ^a^Participants lost to follow-up or did not have complete data required for analyses. ^b^Includes BMI, fat mass, and waist circumference. ^c^Only BMI and fat mass available at this time point. BMI, body mass index; F; female; G0, parent generation 0; G1, offspring generation 1; M, male.

Adiposity measures at 9 y, 15 y, and 18 y were examined in relation to cardiometabolic traits one occasion after (approximately 3 to 6 y later at 15 y, 18 y, and 25 y) in G1. Adiposity measures at 50 y in G0 were examined in relation to cardiometabolic traits also measured at 50 y. To allow full use of measured data, analyses were conducted using maximum numbers of participants (with N varying across ages and between traits). Participants were eligible for inclusion in analyses at any age if they had data on all adiposity measures, sex, and at least one of the cardiometabolic traits plus all relevant potential confounders as listed above. In the G1 cohort, this resulted in 2,190 eligible offspring (1,121 females and 1,069 males) contributing to analysis of adiposity at 9 y and traits at 15 y, 1,600 (813 females and 787 males) contributing to analyses of adiposity at 15 y and traits at 18 y, and 1,613 contributing (928 females and 685 males) to analyses of adiposity at 18 y and traits at 25 y. The single time point analysis in G0 parents included 4,887 eligible parents (3,446 mothers and 1,441 fathers). A flow diagram of the study design is shown in Fig 1.

### Statistical analysis

#### Primary analysis

We standardised all measures of adiposity and all cardiometabolic traits into SD units by generating z scores (subtracting the sex-combined mean and dividing by the SD). Linearity of associations was also examined by comparing models where fifths of adiposity measures were treated as continuous exposures to models where fifths of adiposity measures were treated as categorical exposures using a likelihood ratio (LR) test. Mean outcome traits by fifths of exposure were also plotted and examined to explore substantial deviations from linearity. We found no consistent evidence of departures from linearity for most traits across time points (results from LR tests for key traits shown in S1 Table), and analyses here therefore treat adiposity measures as continuous exposures. Correlations between adiposity measures were also examined, and all 3 measures were highly correlated with each other in each sex at each age with Pearson correlations of greater than 0.8/0.9 between measures.

Linear regression models with robust standard errors to accommodate skewed outcome distributions were used to examine the association between each standardised adiposity measure (BMI, fat mass, and waist circumference) and each standardised cardiometabolic trait. These models included an interaction term for sex and the standardised adiposity measure; this provided the sex-specific mean difference and 95% confidence interval (CI) in each standardised cardiometabolic trait per SD higher adiposity measure. All analyses were performed as described above (inclusive of sex interactions), initially unadjusted, with subsequent adjustment for potential confounders. We also fitted a sex interaction term with all confounding variables separately to allow confounding structures to differ in females and males. As recommended by the American Statistical Association and others [26–28], we focus our descriptions of results on effect size and precision.

Analyses were conducted using Stata 15.1 (Stata, College Station, Texas, USA), and data visualisation was performed in R (version 3.6.3) using the ggforestplot (0.0.2) package. This study is reported as per the Strengthening the Reporting of Observational Studies in Epidemiology (STROBE) guideline (S3 Appendix).

#### Sensitivity and additional analyses

We repeated all analyses using cardiometabolic traits in their original (non-SD) units to aid clinical interpretation. We repeated our analysis using adiposity measures that were standardised using the sex-specific mean and SD to examine whether results were appreciably different to our main analytic approach (which used sex-combined means and SDs for standardisation). We repeated all analyses on the sample whose full family units were eligible for inclusion in all analyses, i.e., offspring had to be eligible for inclusion in each of the 3 G1 analyses and mothers and fathers had to be eligible for inclusion in the G0 analysis. We repeated our analyses excluding participants in the top fifth of the adiposity distribution at each time point to exclude the possibility of results reflecting threshold effects of adiposity measures on cardiometabolic risk. In order to examine potential selection bias in our analysis of G1, we compared characteristics of the G1 participants included in our analyses (*n* = 3,081) compared with G1 participants excluded from analyses by sex. Similarly, we compared characteristics of G0 participants included in our analyses (*n* = 4,887) with G0 participants excluded from analyses by sex. For both G1 and G0 analyses comparing included and excluded participants, we used maternal and paternal sociodemographic and health characteristics measured at or close to birth (at the commencement of the cohort in 1991/1992 when missing data for most characteristics was rarest).

## Results

### Characteristics of included versus excluded participants

G1 offspring included in the analysis had a smaller proportion of UK ethnic minorities and had parents with higher educational attainment, higher household social class, and mothers with lower levels of smoking during pregnancy compared with offspring excluded from analyses (S2 Table). G1 offspring included had older mothers during pregnancy compared with offspring excluded from analyses. G1 included and excluded participants were similar in birth weight, gestational age, and adiposity at each age.

G0 parents included in analyses had a smaller proportion of UK ethnic minorities, were more educated, had higher social class, lower levels of smoking during pregnancy, and were less adipose than parents excluded from analyses (S3 Table).

### Characteristics of participants included in analyses by sex and generation

A minority of male G1 participants and female G1 participants (each <5.0%) were of UK ethnic minority (Table 1). Male G1 participants had parents with slightly higher education, lower levels of smoking among their mothers during pregnancy, higher household social class, and higher birth weight but similar gestational age and maternal age compared with female G1 participants. Male G1 participants had similar BMI and waist circumference but lower fat mass at each age compared with female participants.

A low proportion of G0 fathers and mothers (each <5.0%) were of UK ethnic minority. G0 fathers had higher educational levels and social class and higher levels of smoking during their partners’ pregnancy compared with G0 mothers. G0 fathers had higher BMI and waist circumference but lower fat mass compared with G0 mothers.

**Table 1:** Characteristics of participants included in analyses by sex.

	G1		G0	
	Females *n* = 1,629	Males *n* = 1,452	Females *n* = 3,446	Males *n* = 1,441
	*n* (%)	*n* (%)	*n* (%)	*n* (%)
**UK ethnic minorities**	25 (1.7)	25 (1.5)	64 (1.9)	17 (1.2)
**Maternal education** ^**a**^				
CSE	152 (9.3)	111 (7.6)	273 (7.9)	-
Vocational	104 (6.4)	96 (6.6)	233 (6.8)	-
O level	551 (32.8)	478 (32.9)	1,188 (34.5)	-
A level	479 (29.4)	465 (32.0)	1,064 (30.9)	-
Degree	343 (21.1)	302 (20.8)	688 (20.0)	-
**Paternal education** ^**a**^				
CSE	256 (15.7)	182 (12.5)	-	136 (9.4)
Vocational	113 (6.9)	109 (7.5)	-	78 (5.4)
O level	337 (20.7)	327 (22.5)	-	300 (20.8)
A level	497 (30.5)	416 (28.7)	-	420 (29.2)
Degree	426 (26.2)	418 (28.8)	-	507 (35.2)
**Smoking in pregnancy** ^**b**^	225 (13.8)	186 (12.8)	457 (13.3)	253 (17.6)
**Social class** ^**c**^				
Professional	304 (18.6)	315 (21.7)	280 (8.1)	278 (19.3)
Managerial and technical	772 (47.4)	717 (49.4)	1,318 (38.3)	619 (43.0)
Nonmanual	404 (24.8)	315 (21.7)	1,378 (40.0)	170 (11.8)
Manual	111 (6.8)	82 (5.7)	202 (5.9)	275 (19.1)
Part skilled and unskilled	38 (2.3)	23 (1.6)	268 (7.8)	99 (6.9)
	***Mean (SD)***	***Mean (SD)***	***Mean (SD)***	***Mean (SD)***
**BMI (kg/m** ^**2**^ **) at 9 y**	17.7 (2.8)	17.3 (2.5)	-	-
**Fat mass (kg) at 9 y**	9.4 (4.8)	7.2 (4.6)	-	-
**WC (cm) at 9 y**	62.1 (7.3)	62.8 (7.1)	-	-
**BMI (kg/m** ^**2**^ **) at 15 y**	21.7 (3.5)	20.9 (3.2)	-	-
**Fat mass (kg) at 15 y**	18.7 (8.0)	11.0 (8.0)	-	-
**WC (cm) at 15 y**	76.1 (8.4)	76.7 (8.6)	-	-
**BMI (kg/m** ^**2**^ **) at 18 y**	22.7 (4.0)	22.4 (3.7)	-	-
**Fat mass (kg) at 18 y**	21.1 (8.9)	13.4 (9.6)	-	-
**BMI (kg/m** ^**2**^ **) at 50 y**	-	-	26.4 (5.1)	27.4 (3.9)
**Fat mass (kg) at 50 y**	-	-	26.7 (10.6)	23.4 (8.9)
**WC (cm) at 50 y**	-	-	84.0 (12.0)	97.2 (10.5)
**Birth weight (kg)**	3.39 (0.48)	3.48 (0.57)	-	-
**Gestational age (weeks)**	39.6 (1.6)	39.4 (1.8)	-	-
**Maternal age (years)**	29.4 (4.6)	29.7 (4.4)	-	-

Participants described are those with data on sex, age, BMI, fat mass, at least 1 cardiometabolic trait at any age (for G1) and at age 50 (for G0), and all confounders.

^a^Education was categorised as below O level (exams taken in different subjects usually at age 15 to 16 at the completion of legally required school attendance, equivalent to today’s UK General Certificate of Secondary Education), O level only, A level (exams taken in different subjects usually at age 18), or university degree or above.

^b^Smoking during pregnancy is defined as the mother having self-reported smoking any type of tobacco in the first trimester. For males in G0, smoking during pregnancy is defined as the mother having reported on behalf of the father that the father currently smokes any type of tobacco based on a questionnaire at 18 weeks gestation.

^c^For G1, social class is highest of the mother and father social class. For G0, social class is own social class for the participant.

A level, advanced level; BMI, body mass index; CSE, Certificate of Secondary Education; FM, fat mass; G0, parent generation 0; G1, offspring generation 1; O level, ordinary level; SD, standard deviation; WC, waist circumference.

### Associations of adiposity measures with lipoprotein concentrations in females and males

**Fig 2:**
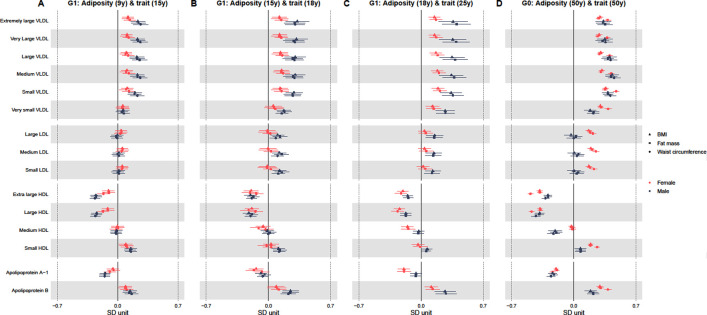
Sex-specific association of BMI, fat mass, and waist circumference (per SD increase) with standardised lipoprotein concentrations from childhood to midlife. Results shown are standardised differences with whiskers representing 95% CIs. These represent the standardised difference in cardiometabolic trait per SD increase in BMI, fat mass, and waist circumference in each sex separately for associations of adiposity at 9 y and traits at 15 y **(A)**, adiposity at 15 y and traits at 18 y **(B)**, adiposity at 18 y and traits at 25 y **(C)**, and adiposity at 50 y and traits at 50 y **(D)**. G1 analyses are adjusted for age at clinic completion, ethnicity, child’s mother and father education, maternal smoking during pregnancy, birth weight, gestational age, maternal age, household social class, and height and height^2^. Analyses of outcomes at 18 y and 25 y are also additionally adjusted for G1 offspring smoking. G0 analyses are adjusted for age at clinic completion, ethnicity, education, smoking during G1 cohort pregnancy, own social class, and height and height^2^. SD unit of BMI = 2.7 kg/m^2^, 2.9 kg/m^2^, 3.8 kg/m^2^, and 4.8 kg/m^2^ at 9 y, 15 y, 18 y, and 50 y, respectively. SD unit of fat mass = 5 kg, 7.8 kg, 9.8 kg, and 10.2 kg at 9 y, 15 y, 18 y, and 50 y, respectively. SD unit of waist circumference = 7.4 cm, 7.8 cm, and 13 cm at 9 y, 15 y, and 50 y, respectively. BMI, body mass index; CI, confidence interval; G0, parent generation 0; G1, offspring generation 1; HDL, high-density lipoprotein; LDL, low-density lipoprotein; SD, standard deviation; VLDL, very-low-density lipoprotein.

**Fig 3:**
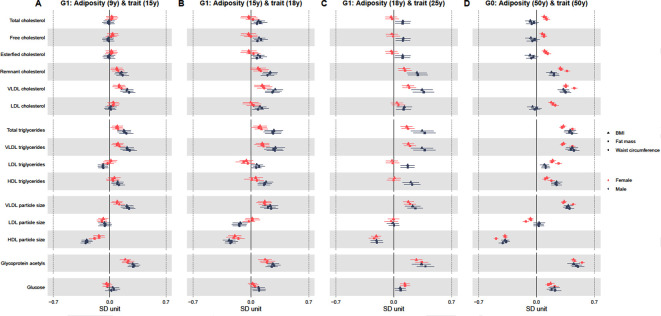
Sex-specific association of in BMI, fat mass, and waist circumference (per SD increase) with standardised cholesterol, triglyceride, and other trait concentrations from childhood to midlife. Results shown are standardised differences with whiskers representing 95% CIs. These represent the standardised difference in cardiometabolic trait per SD increase in BMI, fat mass, and waist circumference in each sex separately for associations of adiposity at 9 y and traits at 15 y **(A)**, adiposity at 15 y and traits at 18 y **(B)**, adiposity at 18 y and traits at 25 y **(C)**, and adiposity at 50 y and traits at 50 y **(D)**. G1 analyses are adjusted for age at clinic completion, ethnicity, child’s mother and father education, maternal smoking during pregnancy, birth weight, gestational age, maternal age, household social class, and height and height^2^. Analyses of outcomes at 18 y and 25 y are also additionally adjusted for G1 offspring smoking. G0 analyses are adjusted for age at clinic completion, ethnicity, education, smoking during G1 cohort pregnancy, own social class, and height and height^2^. SD unit of BMI = 2.7 kg/m^2^, 2.9 kg/m^2^, 3.8 kg/m^2^, and 4.8 kg/m^2^ at 9 y, 15 y, 18 y, and 50 y, respectively. SD unit of fat mass = 5 kg, 7.8 kg, 9.8 kg, and 10.2 kg at 9 y, 15 y, 18 y, and 50 y, respectively. SD unit of waist circumference = 7.4 cm, 7.8 cm, and 13 cm at 9 y, 15 y, and 50 y, respectively. BMI, body mass index; CI, confidence interval; G0, parent generation 0; G1, offspring generation 1; HDL, high-density lipoprotein; LDL, low-density lipoprotein; SD, standard deviation; VLDL, very-low-density lipoprotein.

Tables 2–5 and Figs 2 and 3 show adjusted sex-specific associations of BMI, fat mass, and waist circumference with key cardiometabolic traits at each age, with corresponding *P* values for sex differences in associations also shown in Tables 2–5. Adjusted sex-specific associations and sex differences in associations at each age for all 148 traits are shown in S4–S6 Tables. Results with outcome traits in original units are shown in S7–S9 Tables. Unadjusted results are shown in S10–S12 Tables.

In confounder adjusted analyses (G1 adjusted for age at clinic completion, ethnicity, child’s parents education, maternal smoking during pregnancy, birth weight, gestational age, maternal age, household social class, height, height^2^ and offspring smoking; G0 adjusted for age at clinic completion, ethnicity, education, smoking during G1 cohort pregnancy, own social class, height, and height^2^), adiposity measures (BMI, fat mass, and waist circumference) were positively associated with all concentrations of very-low-density lipoprotein (VLDL) particles in both sexes; associations at 15 y, 18 y, and 25 y were generally stronger in males but more similar between the sexes at 50 y (Fig 2 and Tables 2–5; note that S4–S6 Tables show full results of sex-specific associations and sex differences in associations). For instance, a 1 SD higher BMI at 18 y was associated with 0.36 SD (95% CI = 0.20, 0.52) higher concentrations of extremely large VLDL particles at 25 y in males compared with 0.15 SD (95% CI = 0.09, 0.21) in females, *P* value for sex difference = 0.02 (Table 4). By contrast, at age 50 y, a 1 SD higher BMI was associated with 0.33 SD (95% CI = 0.25, 0.42) and 0.30 SD (95% CI = 0.26, 0.33) higher concentrations of extremely large VLDL particles in males and females, respectively, *P* value for sex difference = 0.42 (Table 5).

**Table 2:** Adjusted sex-specific associations of adiposity measures (per SD increase) at 9 y with standardised lipoprotein, cholesterol, triglyceride, and other trait concentrations at 15 y.

	G1: BMI (9 y) and trait (15 y)		*P* value^b^	G1: FM (9 y) and trait (15 y)		*P* value^b^	G1: WC (9 y) and trait (15 y)		*P* value^b^
Trait^a^	Female	Male		Female	Male		Female	Male	
Extremely large VLDL	0.12 (0.05, 0.18)	0.23 (0.15, 0.32)	0.03	0.12 (0.05, 0.19)	0.24 (0.16, 0.32)	0.03	0.14 (0.07, 0.21)	0.26 (0.18, 0.35)	0.02
Very large VLDL	0.11 (0.04, 0.17)	0.23 (0.15, 0.31)	0.02	0.10 (0.03, 0.17)	0.24 (0.15, 0.32)	0.01	0.13 (0.06, 0.19)	0.26 (0.17, 0.35)	0.01
Large VLDL	0.10 (0.04, 0.16)	0.22 (0.14, 0.31)	0.02	0.09 (0.02, 0.16)	0.23 (0.14, 0.31)	0.01	0.12 (0.05, 0.19)	0.25 (0.17, 0.34)	0.01
Medium VLDL	0.11 (0.04, 0.17)	0.23 (0.15, 0.31)	0.02	0.10 (0.02, 0.17)	0.23 (0.15, 0.31)	0.01	0.13 (0.06, 0.20)	0.26 (0.17, 0.34)	0.02
Small VLDL	0.11 (0.04, 0.18)	0.20 (0.12, 0.28)	0.10	0.10 (0.02, 0.17)	0.19 (0.12, 0.27)	0.07	0.13 (0.06, 0.21)	0.23 (0.15, 0.31)	0.07
Very small VLDL	0.06 (−0.01, 0.13)	0.06 (−0.01, 0.13)	0.97	0.05 (−0.02, 0.13)	0.05 (−0.02, 0.12)	0.93	0.06 (−0.01, 0.14)	0.07 (0.0002, 0.14)	0.87
Large LDL	0.04 (−0.03, 0.11)	−0.01 (−0.07, 0.06)	0.35	0.04 (−0.03, 0.11)	−0.02 (−0.09, 0.05)	0.18	0.03 (−0.04, 0.1)	−0.01 (−0.07, 0.06)	0.45
Medium LDL	0.05 (−0.01, 0.12)	0.01 (−0.06, 0.08)	0.40	0.06 (−0.01, 0.13)	0.002 (−0.07, 0.07)	0.21	0.04 (−0.03, 0.11)	0.01 (−0.06, 0.08)	0.49
Small LDL	0.05 (−0.01, 0.12)	0.01 (−0.06, 0.08)	0.43	0.06 (−0.01, 0.13)	−0.003 (−0.07, 0.07)	0.22	0.05 (−0.02, 0.12)	0.01 (−0.06, 0.08)	0.52
Very large HDL	−0.11 (−0.18, −0.04)	−0.25 (−0.32, −0.19)	0.002	−0.10 (−0.18, −0.03)	−0.25 (−0.31, −0.19)	0.002	−0.17 (−0.24, −0.09)	−0.26 (−0.32, −0.21)	0.03
Large HDL	−0.12 (−0.19, −0.05)	−0.24 (−0.30, −0.18)	0.01	−0.12 (−0.19, −0.04)	−0.25 (−0.31, −0.18)	0.01	−0.17 (−0.24, −0.1)	−0.26 (−0.32, −0.2)	0.05
Medium HDL	−0.004 (−0.08, 0.07)	−0.01 (−0.08, 0.05)	0.83	0.003 (−0.07, 0.08)	−0.02 (−0.09, 0.04)	0.60	−0.02 (−0.09, 0.05)	−0.02 (−0.08, 0.04)	0.99
Small HDL	0.09 (0.02, 0.17)	0.15 (0.08, 0.22)	0.25	0.10 (0.02, 0.18)	0.14 (0.07, 0.21)	0.39	0.11 (0.04, 0.18)	0.15 (0.09, 0.22)	0.34
Apolipoprotein A-I	−0.06 (−0.13, 0.01)	−0.15 (−0.21, −0.09)	0.05	−0.05 (−0.12, 0.02)	−0.15 (−0.22, −0.09)	0.03	−0.09 (−0.16, −0.02)	−0.15 (−0.21, −0.09)	0.19
Apolipoprotein B	0.09 (0.02, 0.16)	0.14 (0.07, 0.22)	0.32	0.09 (0.01, 0.16)	0.13 (0.06, 0.21)	0.37	0.10 (0.03, 0.18)	0.16 (0.08, 0.24)	0.27
Serum total cholesterol	0.02 (−0.04, 0.09)	−0.01 (−0.08, 0.06)	0.49	0.03 (−0.04, 0.10)	−0.02 (−0.09, 0.05)	0.25	0.02 (−0.05, 0.09)	−0.01 (−0.08, 0.06)	0.61
Free cholesterol	0.03 (−0.03, 0.10)	−0.02 (−0.09, 0.05)	0.28	0.04 (−0.03, 0.11)	−0.03 (−0.09, 0.04)	0.16	0.02 (−0.05, 0.09)	−0.02 (−0.08,0.05)	0.49
Esterified cholesterol	0.02 (−0.05, 0.09)	−0.004 (−0.07, 0.06)	0.62	0.03 (−0.04, 0.10)	−0.02 (−0.09, 0.05)	0.31	0.02 (−0.06, 0.09)	−0.01 (−0.07, 0.06)	0.68
Remnant cholesterol	0.09 (0.02, 0.16)	0.15 (0.07, 0.22)	0.26	0.09 (0.01, 0.17)	0.13 (0.06, 0.21)	0.40	0.11 (0.03, 0.18)	0.16 (0.09, 0.23)	0.31
Cholesterol in VLDL	0.12 (0.04, 0.19)	0.22 (0.14, 0.29)	0.07	0.11 (0.04, 0.19)	0.21 (0.13, 0.29)	0.08	0.15 (0.07, 0.22)	0.24 (0.16, 0.32)	0.08
Cholesterol in LDL	0.04 (−0.02, 0.11)	0.01 (−0.05, 0.08)	0.55	0.05 (−0.02, 0.12)	−0.002 (−0.07, 0.07)	0.27	0.04 (−0.03, 0.11)	0.01 (−0.06, 0.08)	0.56
Serum total triglycerides	0.10 (0.03, 0.16)	0.18 (0.10, 0.26)	0.13	0.08 (0.01, 0.16)	0.18 (0.10, 0.26)	0.07	0.10 (0.03, 0.17)	0.21 (0.12, 0.29)	0.06
Triglycerides in VLDL	0.10 (0.04, 0.17)	0.22 (0.13, 0.3)	0.03	0.09 (0.02, 0.16)	0.22 (0.14, 0.3)	0.02	0.12 (0.05, 0.19)	0.25 (0.16, 0.33)	0.02
Triglycerides in LDL	0.01 (−0.06, 0.09)	−0.08 (−0.15, −0.02)	0.05	0.002 (−0.08, 0.08)	−0.09 (−0.15, −0.02)	0.07	−0.02 (−0.10, 0.05)	−0.08 (−0.14, −0.02)	0.20
Triglycerides in HDL	0.06 (−0.02, 0.13)	0.10 (0.03, 0.17)	0.45	0.04 (−0.05, 0.12)	0.09 (0.02, 0.17)	0.28	0.04 (−0.04, 0.12)	0.11 (0.04, 0.19)	0.15
VLDL particle size	0.09 (0.03, 0.16)	0.21 (0.14, 0.29)	0.02	0.09 (0.02, 0.15)	0.22 (0.14, 0.3)	0.01	0.12 (0.05, 0.19)	0.24 (0.16, 0.32)	0.01
LDL particle size	−0.08 (−0.14, −0.02)	−0.07 (−0.14, 0.01)	0.74	−0.10 (−0.16, −0.04)	−0.05 (−0.13, 0.02)	0.31	−0.10 (−0.15, −0.04)	−0.06 (−0.14, 0.01)	0.49
HDL particle size	−0.13 (−0.20, −0.07)	−0.29 (−0.35, −0.22)	0.001	−0.13 (−0.2, −0.06)	−0.28 (−0.35, −0.22)	0.001	−0.19 (−0.26, −0.12)	−0.3 (−0.36, −0.24)	0.01
Glycoprotein acetyls	0.19 (0.13, 0.26)	0.29 (0.22, 0.36)	0.05	0.23 (0.16, 0.30)	0.31 (0.24, 0.37)	0.11	0.22 (0.15, 0.29)	0.29 (0.23, 0.36)	0.13
Glucose	−0.04 (−0.09, 0.01)	0.04 (−0.04, 0.11)	0.09	−0.03 (−0.08, 0.03)	0.05 (−0.02, 0.12)	0.08	−0.03 (−0.09, 0.03)	0.02 (−0.06, 0.09)	0.26

^a^Analysed in SD units.

^b^*P* values shown are for sex differences in estimates.

Detailed sex difference results, results for all other traits, and *P* values corresponding to sex-specific estimates are shown in S4–S6 Tables. Results in original units are shown in S7–S9 Tables, and unadjusted results can be found in S10–S12 Tables. Analyses are adjusted for age at clinic completion, ethnicity, child’s mother and father education, maternal smoking during pregnancy, birth weight, gestational age, maternal age, household social class, and height and height^2^. SD unit of BMI = 2.7 kg/m^2^, 2.9 kg/m^2^, 3.8 kg/m^2^, and 4.8 kg/m^2^ at 9 y, 15 y, 18 y, and 50 y, respectively. SD unit of fat mass = 5 kg, 7.8 kg, 9.8 kg, and 10.2 kg at 9 y, 15 y, 18 y, and 50 y, respectively. SD unit of waist circumference = 7.4 cm, 7.8 cm, and 13 cm at 9 y, 15 y, and 50 y, respectively.

BMI, body mass index; FM, fat mass; G1, offspring generation 1; HDL, high-density lipoprotein; LDL, low-density lipoprotein; SD, standard deviation; VLDL, very-low-density lipoprotein; WC, waist circumference.

**Table 3:** Adjusted sex-specific associations of adiposity measures (per SD increase) at 15 y with standardised lipoprotein, cholesterol, triglyceride, and other trait concentrations at 18 y.

	G1: BMI (15 y) and trait (18 y)		*P* value^b^	G1: FM (15 y) and trait (18 y)		*P* value^b^	G1: WC (15 y) and trait (18 y)		*P* value^b^
Trait^a^	Female	Male		Female	Male		Female	Male	
Extremely large VLDL	0.12 (0.04, 0.20)	0.32 (0.19, 0.46)	0.01	0.13 (0.03, 0.22)	0.30 (0.18, 0.41)	0.03	0.15 (0.07, 0.22)	0.30 (0.19, 0.41)	0.02
Very large VLDL	0.13 (0.05, 0.20)	0.31 (0.19, 0.44)	0.01	0.12 (0.03, 0.21)	0.29 (0.17, 0.4)	0.03	0.14 (0.07, 0.22)	0.30 (0.19, 0.41)	0.02
Large VLDL	0.14 (0.06, 0.21)	0.30 (0.18, 0.42)	0.02	0.13 (0.04, 0.22)	0.27 (0.16, 0.39)	0.05	0.15 (0.08, 0.23)	0.29 (0.19, 0.40)	0.03
Medium VLDL	0.15 (0.07, 0.23)	0.30 (0.19, 0.41)	0.03	0.15 (0.05, 0.24)	0.27 (0.17, 0.38)	0.07	0.16 (0.09, 0.24)	0.29 (0.19, 0.39)	0.04
Small VLDL	0.13 (0.05, 0.22)	0.29 (0.19, 0.38)	0.02	0.13 (0.03, 0.23)	0.27 (0.17, 0.37)	0.05	0.15 (0.07, 0.23)	0.27 (0.19, 0.36)	0.04
Very small VLDL	0.06 (−0.04, 0.15)	0.17 (0.10, 0.25)	0.05	0.06 (−0.05, 0.17)	0.18 (0.10, 0.26)	0.08	0.08 (−0.01, 0.16)	0.15 (0.08, 0.22)	0.19
Large LDL	−0.01 (−0.11, 0.08)	0.10 (0.03, 0.18)	0.06	−0.01 (−0.11, 0.10)	0.13 (0.05, 0.21)	0.04	0.02 (−0.06, 0.11)	0.08 (0.01, 0.15)	0.26
Medium LDL	−0.004 (−0.10, 0.09)	0.12 (0.05, 0.20)	0.04	0.0001 (−0.11,0.11)	0.15 (0.07, 0.23)	0.03	0.03 (−0.05, 0.11)	0.10 (0.03, 0.17)	0.18
Small LDL	−0.01 (−0.11, 0.08)	0.13 (0.05, 0.20)	0.02	−0.01 (−0.12, 0.09)	0.15 (0.07, 0.23)	0.02	0.03 (−0.06,0.11)	0.11 (0.04, 0.18)	0.13
Very large HDL	−0.20 (−0.28, −0.11)	−0.20 (−0.27, −0.14)	0.88	−0.21 (−0.30, −0.11)	−0.17 (−0.24, −0.1)	0.52	−0.14 (−0.22, −0.06)	−0.19 (−0.25, −0.13)	0.33
Large HDL	−0.19 (−0.27, −0.11)	−0.22 (−0.29, −0.15)	0.52	−0.22 (−0.32, −0.13)	−0.19 (−0.25, −0.12)	0.51	−0.15 (−0.23, −0.07)	−0.20 (−0.26, −0.14)	0.29
Medium HDL	−0.06 (−0.14, 0.02)	−0.01 (−0.09, 0.06)	0.40	−0.11 (−0.21, −0.02)	0.02 (−0.06, 0.09)	0.04	−0.03 (−0.12, 0.06)	−0.002 (−0.07, 0.07)	0.58
Small HDL	0.03 (−0.06, 0.12)	0.11 (0.04, 0.19)	0.15	−0.02 (−0.12, 0.08)	0.13 (0.06, 0.21)	0.02	0.03 (−0.07, 0.12)	0.12 (0.05, 0.19)	0.13
Apolipoprotein A-I	−0.14 (−0.22, −0.06)	−0.08 (−0.15, −0.02)	0.30	−0.16 (−0.25, −0.07)	−0.04 (−0.11, 0.03)	0.04	−0.07 (−0.15, 0.01)	−0.07 (−0.13, −0.1)	0.88
Apolipoprotein B	0.09 (−0.01, 0.18)	0.25 (0.16, 0.34)	0.01	0.10 (−0.01, 0.2)	0.25 (0.15, 0.34)	0.04	0.12 (0.03, 0.2)	0.22 (0.14, 0.31)	0.07
Serum total cholesterol	−0.03 (−0.12, 0.06)	0.09 (0.02, 0.17)	0.05	−0.02 (−0.12, 0.08)	0.12 (0.04, 0.19)	0.04	0.02 (−0.06,0.11)	0.08 (0.01, 0.14)	0.35
Free cholesterol	−0.03 (−0.12, 0.06)	0.10 (0.02, 0.17)	0.03	−0.02 (−0.13, 0.08)	0.12 (0.04,0.20)	0.02	−0.001 (−0.08, 0.08)	0.08 (0.01, 0.15)	0.13
Esterified cholesterol	−0.02 (−0.11, 0.06)	0.09 (0.01, 0.16)	0.06	−0.02 (−0.12, 0.08)	0.11 (0.03, 0.19)	0.05	0.03 (−0.05,0.12)	0.07 (0.002, 0.14)	0.45
Remnant cholesterol	0.09 (−0.01, 0.18)	0.23 (0.15, 0.32)	0.02	0.10 (−0.01, 0.21)	0.23 (0.14, 0.32)	0.09	0.12 (0.03, 0.21)	0.20 (0.12, 0.28)	0.17
Cholesterol in VLDL	0.14 (0.05, 0.23)	0.29 (0.20, 0.39)	0.02	0.15 (0.05, 0.26)	0.27 (0.17, 0.37)	0.1	0.16 (0.08, 0.25)	0.26 (0.17, 0.35)	0.10
Cholesterol in LDL	−0.003 (−0.10, 0.09)	0.11 (0.03, 0.19)	0.07	0.01 (−0.1, 0.12)	0.13 (0.05, 0.21)	0.07	0.03 (−0.05, 0.12)	0.09 (0.02, 0.16)	0.33
Serum total triglycerides	0.11 (0.03, 0.19)	0.28 (0.17, 0.39)	0.02	0.10 (0.003, 0.19)	0.26 (0.16, 0.37)	0.02	0.13 (0.05, 0.21)	0.27 (0.17, 0.37)	0.02
Triglycerides in VLDL	0.14 (0.06, 0.22)	0.30 (0.19, 0.41)	0.02	0.13 (0.04, 0.22)	0.27 (0.16, 0.38)	0.05	0.15 (0.08, 0.23)	0.29 (0.19, 0.39)	0.03
Triglycerides in LDL	−0.06 (−0.14, 0.03)	0.07 (−0.002, 0.14)	0.02	−0.09 (−0.18, 0.01)	0.10 (0.03, 0.17)	0.002	−0.04 (−0.12, 0.04)	0.06 (0.003, 0.13)	0.05
Triglycerides in HDL	0.06 (−0.03, 0.14)	0.18 (0.10, 0.27)	0.04	0.01 (−0.08, 0.11)	0.17 (0.08, 0.25)	0.02	0.07 (−0.01, 0.16)	0.16 (0.08, 0.24)	0.13
VLDL particle size	0.17 (0.09, 0.25)	0.23 (0.14, 0.33)	0.29	0.16 (0.07, 0.25)	0.21 (0.11, 0.3)	0.47	0.17 (0.10, 0.25)	0.25 (0.16, 0.33)	0.20
LDL particle size	0.02 (−0.06, 0.10)	−0.13 (−0.22, −0.04)	0.02	0.02 (−0.07, 0.11)	−0.14 (−0.23, −0.05)	0.01	−0.02 (−0.10, 0.05)	−0.14 (−0.22, −0.06)	0.04
HDL particle size	−0.19 (−0.27, −0.12)	−0.26 (−0.34, −0.19)	0.20	−0.21 (−0.29, −0.12)	−0.23 (−0.31, −0.16)	0.64	−0.15 (−0.23, −0.07)	−0.25 (−0.31, −0.18)	0.06
Glycoprotein acetyls	0.17 (0.09, 0.25)	0.27 (0.19, 0.34)	0.10	0.20 (0.11, 0.29)	0.28 (0.2, 0.36)	0.18	0.19 (0.12, 0.27)	0.25 (0.18, 0.32)	0.26
Glucose	0.02 (−0.03, 0.07)	0.09 (0.02, 0.17)	0.13	0.03 (−0.02, 0.09)	0.10 (0.02, 0.18)	0.19	0.05 (−0.002, 0.10)	0.10 (0.03, 0.17)	0.27

^a^Analysed in SD units.

^b^*P* values shown are for sex differences in estimates.

Detailed sex difference results, results for all other traits, and *P* values corresponding to sex-specific estimates are shown in S4–S6 Tables. Results in original units are shown in S7–S9 Tables, and unadjusted results can be found in S10–S12 Tables. Analyses are adjusted for age at clinic completion, ethnicity, child’s mother and father education, maternal smoking during pregnancy, birth weight, gestational age, maternal age, household social class, and height and height^2^ and offspring smoking. SD unit of BMI = 2.7 kg/m^2^, 2.9 kg/m^2^, 3.8 kg/m^2^, and 4.8 kg/m^2^ at 9 y, 15 y, 18 y, and 50 y, respectively. SD unit of fat mass = 5 kg, 7.8 kg, 9.8 kg, and 10.2 kg at 9 y, 15 y, 18 y, and 50 y, respectively. SD unit of waist circumference = 7.4 cm, 7.8 cm, and 13 cm at 9 y, 15 y, and 50 y, respectively.

BMI, body mass index; FM, fat mass; G1, offspring generation 1; HDL, high-density lipoprotein; LDL, low-density lipoprotein; SD, standard deviation; VLDL, very-low-density lipoprotein; WC, waist circumference.

**Table 4:** Adjusted sex-specific associations of adiposity measures (per SD increase) at 18 y with standardised lipoprotein, cholesterol, triglyceride, and other trait concentrations at 25 y.

	G1: BMI (18 y) and trait (25 y)		*P* value^b^	G1: FM (18 y) and trait (25 y)		*P* value^b^
Trait^a^	Female	Male		Female	Male	
Extremely large VLDL	0.15 (0.09, 0.21)	0.36 (0.20, 0.52)	0.02	0.17 (0.10, 0.24)	0.40 (0.24, 0.56)	0.01
Very large VLDL	0.15 (0.08, 0.22)	0.36 (0.20, 0.51)	0.02	0.17 (0.10, 0.24)	0.39 (0.24, 0.54)	0.01
Large VLDL	0.17 (0.10, 0.23)	0.35 (0.21, 0.49)	0.02	0.19 (0.11, 0.26)	0.38 (0.24, 0.52)	0.01
Medium VLDL	0.18 (0.12, 0.25)	0.34 (0.21, 0.48)	0.04	0.20 (0.13, 0.28)	0.37 (0.24, 0.5)	0.03
Small VLDL	0.19 (0.12, 0.26)	0.34 (0.22, 0.46)	0.04	0.21 (0.14, 0.29)	0.36 (0.24, 0.48)	0.05
Very small VLDL	0.13 (0.05, 0.20)	0.27 (0.17, 0.38)	0.03	0.14 (0.06, 0.23)	0.28 (0.17, 0.38)	0.05
Large LDL	0.04 (−0.03, 0.11)	0.15 (0.06, 0.25)	0.06	0.05 (−0.03, 0.13)	0.15 (0.05, 0.24)	0.13
Medium LDL	0.04 (−0.03, 0.11)	0.14 (0.05, 0.23)	0.07	0.06 (−0.02, 0.14)	0.14 (0.04, 0.23)	0.22
Small LDL	0.02 (−0.05, 0.09)	0.13 (0.04, 0.22)	0.06	0.05 (−0.03, 0.13)	0.12 (0.04, 0.21)	0.20
Very large HDL	−0.21 (−0.27, −0.14)	−0.15 (−0.21, −0.09)	0.22	−0.23 (−0.29, −0.16)	−0.15 (−0.20, −0.09)	0.08
Large HDL	−0.24 (−0.30, −0.18)	−0.17 (−0.24, −0.11)	0.11	−0.26 (−0.33, −0.20)	−0.17 (−0.24, −0.11)	0.06
Medium HDL	−0.15 (−0.22, −0.09)	−0.03 (−0.09, 0.04)	0.01	−0.15 (−0.22, −0.07)	−0.03 (−0.09, 0.03)	0.02
Small HDL	−0.04 (−0.11, 0.04)	0.07 (0.01, 0.13)	0.02	−0.01 (−0.09, 0.07)	0.06 (0.003, 0.11)	0.19
Apolipoprotein A-I	−0.19 (−0.26, −0.13)	−0.06 (−0.12, 0.01)	0.004	−0.19 (−0.27, −0.12)	−0.06 (−0.12, 0.004)	0.01
Apolipoprotein B	0.11 (0.04, 0.19)	0.27 (0.15, 0.39)	0.03	0.13 (0.05, 0.21)	0.28 (0.16, 0.40)	0.04
Serum total cholesterol	−0.03 (−0.10, 0.04)	0.11 (0.02, 0.20)	0.01	−0.02 (−0.10, 0.06)	0.11 (0.02, 0.20)	0.04
Free cholesterol	−0.02 (−0.09, 0.05)	0.12 (0.03, 0.21)	0.02	−0.01 (−0.09, 0.07)	0.11 (0.02, 0.20)	0.05
Esterified cholesterol	−0.03 (−0.10, 0.04)	0.11 (0.02, 0.2)	0.02	−0.02 (−0.10, 0.06)	0.11 (0.02, 0.20)	0.04
Remnant cholesterol	0.12 (0.05, 0.20)	0.28 (0.16, 0.4)	0.03	0.14 (0.06, 0.22)	0.29 (0.17, 0.41)	0.03
Cholesterol in VLDL	0.17 (0.10, 0.25)	0.34 (0.21, 0.47)	0.02	0.19 (0.11, 0.27)	0.36 (0.23, 0.49)	0.02
Cholesterol in LDL	0.04 (−0.03, 0.11)	0.13 (0.04, 0.22)	0.12	0.06 (−0.02, 0.14)	0.12 (0.03, 0.21)	0.29
Serum total triglycerides	0.16 (0.09, 0.23)	0.34 (0.21, 0.48)	0.02	0.18 (0.10, 0.25)	0.37 (0.24, 0.51)	0.01
Triglycerides in VLDL	0.18 (0.11, 0.25)	0.34 (0.21, 0.48)	0.03	0.20 (0.12, 0.27)	0.37 (0.24, 0.51)	0.02
Triglycerides in LDL	−0.02 (−0.10, 0.05)	0.17 (0.09, 0.25)	0.001	−0.01 (−0.09, 0.07)	0.17 (0.09, 0.25)	0.002
Triglycerides in HDL	0.01 (−0.06, 0.08)	0.21 (0.11, 0.31)	0.002	−0.003 (−0.08, 0.08)	0.22 (0.13, 0.32)	*P* < 0.001
VLDL particle size	0.18 (0.11, 0.24)	0.23 (0.14, 0.32)	0.37	0.20 (0.13, 0.27)	0.26 (0.17, 0.35)	0.28
LDL particle size	−0.002 (−0.07, 0.07)	−0.01 (−0.08, 0.06)	0.87	−0.03 (−0.11, 0.05)	0.004 (−0.06, 0.07)	0.48
HDL particle size	−0.21 (−0.27, −0.15)	−0.21 (−0.28, −0.14)	0.94	−0.24 (−0.30, −0.17)	−0.21 (−0.28, −0.13)	0.52
Glycoprotein acetyls	0.27 (0.20, 0.34)	0.34 (0.22, 0.45)	0.37	0.34 (0.26, 0.42)	0.38 (0.28, 0.49)	0.52
Glucose	0.13 (0.08, 0.19)	0.08 (0.01, 0.15)	0.22	0.14 (0.08, 0.20)	0.08 (0.02, 0.15)	0.24

^**a**^Analysed in SD units.

^**b**^*P* values shown are for sex differences in estimates.

Detailed sex difference results, results for all other traits, and *P* values corresponding to sex-specific estimates are shown in S4–S6 Tables. Results in original units are shown in S7–S9 Tables, and unadjusted results can be found in S10–S12 Tables. Analyses are adjusted for age at clinic completion, ethnicity, child’s mother and father education, maternal smoking during pregnancy, birth weight, gestational age, maternal age, household social class, and height and height^2^ and offspring smoking. SD unit of BMI = 2.7 kg/m^2^, 2.9 kg/m^2^, 3.8 kg/m^2^, and 4.8 kg/m^2^ at 9 y, 15 y, 18 y, and 50 y, respectively. SD unit of fat mass = 5 kg, 7.8 kg, 9.8 kg, and 10.2 kg at 9 y, 15 y, 18 y, and 50 y, respectively. SD unit of waist circumference = 7.4 cm, 7.8 cm, and 13 cm at 9 y, 15 y, and 50 y, respectively.

BMI, body mass index; FM, fat mass; G1, offspring generation 1; HDL, high-density lipoprotein; LDL, low-density lipoprotein; SD, standard deviation; VLDL, very-low-density lipoprotein; WC, waist circumference.

**Table 5:** Adjusted sex-specific associations of adiposity measures (per SD increase) at 50 y with standardised lipoprotein, cholesterol, triglyceride, and other trait concentrations at 50 y.

	G1: BMI (50 y) and trait (50 y)		*P* value^b^	G1: FM (50 y) and trait (50 y)		*P* value^b^	G1: WC (50 y) and trait (50 y)		*P* value^b^
Trait^a^	Female	Male		Female	Male		Female	Male	
Extremely large VLDL	0.30 (0.26, 0.33)	0.33 (0.25, 0.42)	0.42	0.28 (0.25, 0.31)	0.33(0.25, 0.40)	0.25	0.38 (0.35, 0.42)	0.35 (0.27, 0.44)	0.54
Very large VLDL	0.29 (0.25, 0.32)	0.35 (0.27, 0.44)	0.16	0.27 (0.24, 0.30)	0.32 (0.25, 0.40)	0.18	0.38 (0.34, 0.41)	0.36 (0.27, 0.44)	0.69
Large VLDL	0.30 (0.27, 0.33)	0.40 (0.33, 0.48)	0.02	0.29 (0.26, 0.32)	0.38 (0.32, 0.45)	0.01	0.40 (0.37, 0.44)	0.42 (0.34, 0.49)	0.81
Medium VLDL	0.32 (0.29, 0.35)	0.43 (0.36, 0.51)	0.004	0.31 (0.28, 0.33)	0.42 (0.36, 0.49)	0.001	0.42 (0.39, 0.46)	0.45 (0.38, 0.52)	0.41
Small VLDL	0.36 (0.33, 0.39)	0.38 (0.32, 0.45)	0.51	0.35 (0.32, 0.38)	0.39 (0.33, 0.44)	0.30	0.48 (0.44, 0.51)	0.41 (0.35, 0.48)	0.08
Very small VLDL	0.30 (0.27, 0.33)	0.19 (0.12, 0.25)	0.003	0.31 (0.28, 0.34)	0.22 (0.16, 0.28)	0.02	0.39 (0.35, 0.42)	0.22 (0.15, 0.29)	*P* < 0.001
Large LDL	0.17 (0.13, 0.20)	−0.03 (−0.11, 0.05)	*P* < 0.001	0.19 (0.15, 0.22)	0.03 (−0.04, 0.09)	*P* < 0.001	0.22 (0.18, 0.25)	−0.001 (−0.08, 0.08)	*P* < 0.001
Medium LDL	0.19 (0.16, 0.23)	0.01 (−0.07, 0.08)	*P* < 0.001	0.21 (0.18, 0.24)	0.05 (−0.01, 0.12)	*P* < 0.001	0.25 (0.21, 0.29)	0.04 (−0.04, 0.11)	*P* < 0.001
Small LDL	0.17 (0.14, 0.20)	0.001 (−0.08, 0.08)	*P* < 0.001	0.18 (0.15, 0.21)	0.05 (−0.02, 0.12)	0.001	0.23 (0.19, 0.26)	0.03 (−0.04, 0.11)	*P* < 0.001
Very large HDL	−0.38 (−0.41, −0.35)	−0.29 (−0.33, −0.25)	0.001	−0.38 (−0.41, −0.35)	−0.29 (−0.33, −0.25)	*P* < 0.001	−0.49 (−0.52, −0.46)	−0.32 (−0.36, −0.28)	*P* < 0.001
Large HDL	−0.38 (−0.41, −0.35)	−0.38 (−0.44, −0.33)	0.92	−0.38 (−0.4, −0.35)	−0.39 (−0.44, −0.34)	0.73	−0.48 (−0.51, −0.44)	−0.43 (−0.48, −0.38)	0.10
Medium HDL	−0.03 (−0.06, 0.004)	−0.21 (−0.28, −0.13)	*P* < 0.001	−0.02 (−0.05, 0.02)	−0.20 (−0.27, −0.13)	*P* < 0.001	−0.02 (−0.05, 0.02)	−0.23 (−0.30, −0.16)	*P* < 0.001
Small HDL	0.19 (0.16, 0.22)	0.08 (0.01, 0.14)	0.001	0.19 (0.16, 0.22)	0.08 (0.02, 0.13)	0.001	0.26 (0.23, 0.29)	0.08 (0.02, 0.14)	*P* < 0.001
Apolipoprotein A-I	−0.20 (−0.23, −0.17)	−0.24 (−0.29, −0.19)	0.16	−0.19 (−0.22, −0.17)	−0.22 (−0.27, −0.18)	0.23	−0.24 (−0.27, −0.21)	−0.26 (−0.30, −0.21)	0.53
Apolipoprotein B	0.29 (0.26, 0.33)	0.19 (0.12, 0.26)	0.01	0.30 (0.27, 0.33)	0.22 (0.16, 0.28)	0.01	0.39 (0.35, 0.42)	0.22 (0.15, 0.28)	*P* < 0.001
Serum total cholesterol	0.09 (0.06, 0.13)	−0.08 (−0.14, −0.01)	*P* < 0.001	0.11 (0.08, 0.14)	−0.03 (−0.09, 0.02)	*P* < 0.001	0.12 (0.09, 0.16)	−0.06 (−0.12, 0.004)	*P* < 0.001
Free cholesterol	0.07 (0.04, 0.10)	−0.07 (−0.13, −0.01)	*P* < 0.001	0.09 (0.06, 0.12)	−0.02 (−0.08, 0.04)	0.001	0.09 (0.05, 0.13)	−0.05 (−0.12, 0.01)	*P* < 0.001
Esterified cholesterol	0.10 (0.06, 0.13)	−0.08 (−0.14, −0.02)	*P* < 0.001	0.12 (0.08, 0.15)	−0.04 (−0.09, 0.02)	*P* < 0.001	0.13 (0.10, 0.17)	−0.06 (−0.13, −0.001)	*P* < 0.001
Remnant cholesterol	0.29 (0.25, 0.32)	0.18 (0.12, 0.24)	0.004	0.30 (0.27, 0.33)	0.21 (0.16, 0.27)	0.01	0.37 (0.33, 0.41)	0.21 (0.15, 0.27)	*P* < 0.001
Cholesterol in VLDL	0.35 (0.32, 0.39)	0.32 (0.26, 0.39)	0.43	0.35 (0.32, 0.39)	0.34 (0.28, 0.40)	0.63	0.46 (0.42, 0.49)	0.35 (0.29, 0.42)	0.01
Cholesterol in LDL	0.18 (0.14, 0.21)	−0.05 (−0.11, 0.02)	*P* < 0.001	0.20 (0.16, 0.23)	0.001 (−0.06, 0.06)	*P* < 0.001	0.23 (0.19, 0.27)	−0.02 (−0.09, 0.04)	*P* < 0.001
Serum total triglycerides	0.32 (0.29, 0.36)	0.41 (0.34, 0.48)	0.03	0.31 (0.28, 0.34)	0.40 (0.34, 0.46)	0.01	0.43 (0.40, 0.47)	0.43 (0.36, 0.5)	0.87
Triglycerides in VLDL	0.33 (0.3, 0.36)	0.43 (0.36, 0.5)	0.01	0.31 (0.28, 0.34)	0.42 (0.35, 0.48)	0.003	0.44 (0.40, 0.47)	0.45 (0.38, 0.52)	0.71
Triglycerides in LDL	0.19 (0.16, 0.23)	0.10 (0.05, 0.15)	0.01	0.19 (0.15, 0.22)	0.11 (0.07, 0.16)	0.01	0.27 (0.23, 0.31)	0.11 (0.06, 0.16)	*P* < 0.001
Triglycerides in HDL	0.13 (0.09, 0.16)	0.24 (0.18, 0.31)	0.002	0.11 (0.07, 0.14)	0.23 (0.18, 0.29)	*P* < 0.001	0.18 (0.14, 0.22)	0.24 (0.18, 0.3)	0.09
VLDL particle size	0.33 (0.31, 0.36)	0.38 (0.33, 0.44)	0.11	0.32 (0.30, 0.35)	0.37 (0.32, 0.42)	0.11	0.44 (0.41, 0.47)	0.40 (0.35, 0.45)	0.15
LDL particle size	−0.07 (−0.10, −0.04)	0.03 (−0.04, 0.10)	0.01	−0.07 (−0.10, −0.04)	0.03 (−0.03, 0.08)	0.01	−0.13 (−0.17, −0.09)	0.03 (−0.04, 0.09)	*P* < 0.001
HDL particle size	−0.38 (−0.41, −0.36)	−0.38 (−0.43, −0.33)	0.94	−0.38 (−0.40, −0.35)	−0.37 (−0.41, −0.32)	0.80	−0.49 (−0.52, −0.46)	−0.41 (−0.46, −0.36)	0.004
Glycoprotein acetyls	0.45 (0.42, 0.47)	0.45 (0.38, 0.52)	0.93	0.46 (0.43, 0.49)	0.48 (0.42, 0.55)	0.56	0.55 (0.52, 0.58)	0.50 (0.43, 0.58)	0.23
Glucose	0.17 (0.13, 0.21)	0.22 (0.15, 0.28)	0.27	0.14 (0.11, 0.18)	0.18 (0.12, 0.24)	0.25	0.22 (0.17, 0.27)	0.22 (0.15, 0.30)	0.90

^a^Analysed in SD units.

^b^*P* values shown are for sex differences in estimates.

Detailed sex difference results, results for all other traits, and *P* values corresponding to sex-specific estimates are shown in S4–S6 Tables. Results in original units are shown in S7–S9 Tables, and unadjusted results can be found in S10–S12 Tables. Analyses are adjusted for age at clinic completion, ethnicity, education, smoking during G1 cohort pregnancy, own social class, and height and height^2^. SD unit of BMI = 2.7 kg/m^2^, 2.9 kg/m^2^, 3.8 kg/m^2^, and 4.8 kg/m^2^ at 9 y, 15 y, 18 y, and 50 y, respectively. SD unit of fat mass = 5 kg, 7.8 kg, 9.8 kg, and 10.2 kg at 9 y, 15 y, 18 y, and 50 y, respectively. SD unit of waist circumference = 7.4 cm, 7.8 cm, and 13 cm at 9 y, 15 y, and 50 y, respectively.

BMI, body mass index; FM, fat mass; G1, offspring generation 1; HDL, high-density lipoprotein; LDL, low-density lipoprotein; SD, standard deviation; VLDL, very-low-density lipoprotein; WC, waist circumference.

Adiposity measures were positively associated with all concentrations of low-density lipoprotein (LDL) particles at 18 y and 25 y in males only (Fig 2 and Tables 2–5 and S4–S6). For instance, a 1 SD higher BMI at 15 y was associated with 0.13 SD (95% CI = 0.05, 0.20) higher concentrations of small LDL at 18 y among males compared with a difference of −0.01 SD (95% CI = −0.11, 0.08) among females, *P* value for sex difference = 0.02 (Table 3). At 50 y, adiposity measures were associated with concentrations of LDL in females only (e.g., a 1 SD higher BMI at 50 y was associated with 0.0001 SD (95% CI = −0.08, 0.08) higher concentrations of small LDL among males compared with 0.17 SD (95% CI = 0.14, 0.20) among females, *P* value for sex difference = <0.001 [Table 5]). By contrast, adiposity measures were inversely associated with concentrations of very large and large high-density lipoprotein (HDL) particles at all ages in both sexes; associations were stronger in males at 15 y, but more similar between males and females from 18 y onwards (e.g., a 1 SD higher BMI at 50 y was associated with −0.38 SD (95% CI = −0.44, −0.33) lower concentrations of large HDL at 50 y among males, with similar differences among females, *P* value for sex difference = 0.92 [Table 5]).

Adiposity measures were inversely associated with concentrations of medium HDL particles at 25 y in females only and at 50 y in males only (e.g., a 1 SD higher BMI at 18 y was associated with −0.15 SD (95% CI = −0.22, −0.09) lower concentrations of medium HDL at 25 y among females, while a 1 SD higher BMI at 50 y was associated with −0.21 SD (95% CI = −0.28, −0.13) lower concentrations of medium HDL at 50 y among males [Fig 2 and Tables 2–5 and S4–S6]). Adiposity measures were positively associated with concentrations of small HDL particles at younger ages among males and in both sexes at 50 y; positive associations with small HDL were stronger in females at 50 y (e.g., a 1 SD higher BMI at 50 y was associated with 0.08 SD (95% CI = 0.01,0.14) higher concentrations of small HDL among males at 50 y compared with 0.19 SD (95% CI = 0.16,0.22) among females, *P* value for sex difference = 0.001 [Table 5]).

Adiposity measures were inversely associated with apolipoprotein A-1 and positively associated with apolipoprotein B at each age in both sexes; at younger ages, associations with apolipoprotein A-1 concentrations were stronger in females (e.g., a 1 SD higher BMI at 18 y was associated with −0.06 SD (95% CI = −0.12,0.01) lower concentrations of apolipoprotein A-1 among males at 25 y compared with −0.19 SD (95% CI = −0.26, −0.13) in females, *P* value for difference = 0.004 [Fig 2 and Tables 2–5 and S4–S6]). Associations with apolipoprotein B concentrations were stronger in males at younger ages (e.g., a 1 SD higher BMI at 15 y was associated with 0.25 SD (95% CI = 0.16, 0.34) higher apolipoprotein B concentrations among males at 18 y compared with 0.09 SD (95% CI = −0.01, 0.18) among females, *P* value for sex difference = 0.01 [Table 3]). At 50 y, associations were similar between the sexes for apolipoprotein A-1 (e.g., a 1 SD higher BMI at 50 y was associated with −0.24 SD (95% CI = −0.29, −0.19) lower concentrations of apolipoprotein A-1 among males, with similar differences among females, *P* value for sex difference = 0.16 [Table 5]). By contrast, associations were stronger in females for apolipoprotein B at 50 y (e.g., a 1 SD higher BMI at 50 y was associated with 0.19 SD (95% CI = 0.12, 0.25) higher concentrations of apolipoprotein B at 50 y among males compared with 0.29 SD (95% CI = 0.26, 0.33) in females, *P* value for sex difference = 0.01 [Table 5]).

### Associations of adiposity measures with cholesterol, triglyceride, and other trait concentrations in females and males

Adiposity measures were positively associated with remnant and VLDL cholesterol in both sexes at all ages (Fig 3 and Tables 2–5 and S4–S6); associations were stronger in males at younger ages (e.g., a 1 SD higher BMI at 15 y was associated with 0.29 SD (95% CI = 0.20, 0.39) higher concentrations of VLDL cholesterol in males at 18 y compared with 0.14 SD (95% CI = 0.05, 0.23) among females, *P* value for sex difference = 0.02 [Table 3]). By contrast, associations were more similar between the sexes at 50 y (e.g., a 1 SD higher BMI at 50 y was associated with 0.32 SD (95% CI = 0.26, 0.39) higher concentrations of VLDL cholesterol in males at 50 y with a similar difference in females, *P* value for sex difference = 0.43 [Table 5]). Adiposity measures were positively associated with total, free, esterified, and LDL cholesterol in males only at 18 and 25 y. At 50 y, adiposity measures were inversely associated with total, free, esterified, and LDL cholesterol concentrations in males but positively associated with these in females (e.g., a 1 SD higher BMI at 50 y was associated with −0.05 SD (95% CI = −0.11, 0.02) lower concentrations of LDL cholesterol in males compared with 0.18 SD (95% CI = 0.14, 0.21) higher concentrations of LDL cholesterol among females, *P* value for sex difference = <0.001 [Table 5]).

Adiposity measures were positively associated with total and VLDL triglyceride concentrations at all ages in both sexes; associations were stronger in males at each age (Fig 3 and Tables 2–5 and S4–S6). For instance, a 1 SD higher BMI at 18 y was associated with 0.34 SD (95% CI = 0.21, 0.48,) higher total triglyceride concentrations at 25 y in males compared with 0.16 SD (95% CI = 0.09, 0.23) in females, *P* value for sex difference = 0.02 [Table 4]). Adiposity measures were positively associated with LDL and HDL triglyceride concentrations in males only at 18 and 25 y (e.g., a 1 SD higher BMI at 18 y was associated with 0.17 SD (95% CI = 0.09, 0.25) higher concentrations of LDL triglycerides at 25 y among males compared with −0.02 SD (95% CI = −0.1, 0.05) among females, *P* value for sex difference = 0.001 [Table 4]). Adiposity measures were positively associated with LDL and HDL triglyceride concentrations in both sexes at 50 y, with associations for LDL triglycerides stronger in females and HDL triglycerides stronger in males (e.g., a 1 SD higher BMI at 50 y was associated with 0.24 SD (95% CI = 0.18,0.31) higher HDL triglyceride concentrations among males compared with 0.13 SD (95% CI = 0.09,0.16) among females, *P* value for sex difference = 0.002 [Table 5]).

Adiposity measures were positively associated with VLDL particle size and inversely associated with HDL particle size at all ages; associations were stronger in males compared with females at 15 y and 18 y, while associations were similar between the sexes at 25 y and 50 y (Fig 3 and Tables 2–5 and S4–S6). For instance, a 1 SD higher BMI at 9 y was associated with 0.21 SD (95% CI = 0.14, 0.29) greater VLDL particle size among males at 15 y compared with 0.09 SD (95% CI = 0.03, 0.16) among females, *P* value for sex difference = 0.02 [Table 2]). Adiposity measures were positively associated with glycoprotein acetyls in both sexes at each time point and associations were stronger in males at 15 y and 18 y but more similar between the sexes at 25 y and 50 y. Adiposity measures were similarly positively associated with glucose concentrations in both sexes at 25 y and 50 y (e.g., a 1 SD higher BMI at 50 y was associated with 0.22 SD (95% CI = 0.15, 0.28) higher glucose concentrations in males compared with 0.17 SD (95% CI = 0.13, 0.21) in females, *P* value for sex difference = 0.27 [Table 5]).

Associations of fat mass and waist circumference with traits were broadly similar across each time point (Figs 2 and 3 and Tables 2–5 and S4–S6). Unadjusted and confounder adjusted results were also similar (S10–S12 Tables).

### Additional and sensitivity analyses

Our results were not appreciably different when analyses were performed using the sex-specific mean and SD of each adiposity measure for standardisation (S1 and S2 Figs) or when analyses were restricted to full family units (S3 and S4 Figs). Associations were also similar when participants in the upper fifth of the adiposity distribution were excluded from analyses suggesting that modest departures from linearity for some traits are unlikely to have impacted our results (S5 and S6 Figs).

## Discussion

### Summary

In this UK prospective cohort study including 2 generations of participants, we examined sex-specific associations of general adiposity with detailed cardiometabolic traits measured in mid and late adolescence and early and mid adulthood. Overall, our findings suggest that the associations of adiposity with adverse cardiometabolic risk begin earlier in the life course in males compared with females and are stronger until midlife. Adolescent and young adult males may therefore be high priority targets for obesity prevention efforts. Our findings also demonstrate high consistency between BMI, DXA fat mass, and waist circumference in their ability to detect associations with detailed cardiometabolic traits at each life stage, supporting the continued use of BMI as a simple and inexpensive measure of adiposity for analyses in both sexes across the life course.

### Added value of the research and comparison with previous research

Cardiometabolic disease risk tends to appear higher in males until midlife, after which risk becomes more similar between females and males [5]. In previous ALSPAC studies of the same cardiometabolic traits studied here, absolute levels of several key causal CHD traits were higher in males compared with females in early life and became more similar or lower in males compared with females in midlife [3,29]. Our findings build on this existing research by suggesting that stronger early life and weaker later life associations of adiposity with cardiometabolic traits in males play an important role in these previously identified life course sex differences in cardiometabolic risk [3]. In addition, several of the traits that were higher in males in these studies [3,29] and more strongly associated with adiposity in males in our study such as LDL cholesterol [30], VLDL triglycerides [31], and apolipoprotein B [32–34] are very likely causal for CHD. HDL cholesterol, an important noncausal marker of CHD risk [35] was also lower among males in those studies and more strongly associated with adiposity among males in the present study. If sex differences in associations of adiposity with these cardiometabolic traits found in our study reflect sex differences in causal effects, our findings provide new insights into potential drivers of sex differences in age-adjusted risk of CHD across the life course. The more adverse pattern seen among males, despite their lower total fat than females, in turn likely reflects sex differences in patterns of fat storage with males storing more metabolically harmful abdominal fat and females storing more peripheral fat, which is less metabolically active. This is exemplified by the so-called “favourable adiposity” phenotype, underpinned by a genotype wherein higher fat volume is accompanied by higher insulin sensitivity and better cardiometabolic health [36,37]. While previous studies have demonstrated strong comparability of observational and causal estimates of BMI and metabolites [7], additional study designs including mendelian randomisation are required to interrogate the causality of observed sex-specific associations found here.

While studies examining associations of adiposity with cardiometabolic traits at multiple life stages are limited, our results are comparable with previous work in our [38,39] cohort showing sex-specific associations of adiposity with conventional cardiometabolic risk factors in adolescence to the disadvantage of males. The results are also comparable to findings from a Young Finns analysis of 12,664 adolescents and young adults aged 16 to 39, which demonstrated similar evidence for stronger associations of BMI with causal CHD susceptibility traits in males, using evidence from mendelian randomisation [7]; however, a key limitation of this study was limited statistical power to estimate sex-specific effects with certainty. The similarity of sex-specific associations of BMI, fat mass, and waist circumference with cardiometabolic traits at each life stage in our study is comparable to previous studies in adolescence and adulthood [14,38], although some analyses in adults have reported differences in associations [15]. Differences in findings in comparison with previous work may include a focus on examining and comparing associations of adiposity with CVD end points, often at single time points in adulthood, where reverse causation is more likely than when studying metabolic trait concentrations as we have done here.

### Strengths and limitations

There are several strengths to our study including the use of both general and central measures of adiposity and the direct comparison of the associations of these measures with approximately 148 detailed cardiometabolic trait concentrations from a targeted metabolomics platform measured at multiple life stages. The NMR spectroscopy technique used here is also almost entirely free from batch effects because the samples never come into contact with the radiofrequency detector in the NMR spectrometer [40,41]. In addition, we have performed multiple sensitivity analyses including examining the robustness of our findings to standardisation of the exposures using sex-specific mean and SD, to potential selection issues (by performing analyses only on mother–father–offspring trios), and to threshold effects of adiposity on outcomes (by excluding the upper fifth of the adiposity distribution).

Limitations include missing data and loss to follow-up, leading to modest sample sizes for inclusion in analyses compared with the numbers available in the originally recruited birth cohort. If loss to follow-up is differentially related to both exposure and outcome by sex, estimates of sex-specific associations at each life stage could be biased. In addition, estimates of sex-specific associations in the G1 cohort compared with the G0 parent cohort could be further undermined by different degrees of selection bias in the G1 and G0 cohorts. For instance, although we found demographic characteristics were comparable between included and excluded females and males in each cohort separately, we did find some evidence of greater sex-specific selection in the G0 cohort, which may impact the validity of the comparison of the 2 cohorts. Furthermore, while measures analysed in G1 cohort analyses were prospective, analyses at 50 y were cross-sectional. Importantly, we cannot exclude the possibility that differences in estimates between the 2 cohorts are not a product of “cohort effects” driven by generational differences between the children and their parents such as greater exposure of the G1 cohort to the current obesity epidemic. While we included and compared 3 general measures of adiposity here, the measures do not capture fine scale adiposity and adiposity distribution differences in females and males including shifts towards “metabolically favourable adiposity” [36], which may explain less adverse associations of adiposity among females found here. Residual confounding may also persist in some of our analyses due to lack of adjustment for other confounders such as alcohol use or medical conditions, particularly in the G0 parent cohort. Finally, participants were predominantly of White ethnicity and more socially advantaged, and thus, our results may not be generalisable to other populations.

### Implications

In this UK prospective cohort study including 2 generations of participants, we demonstrated that adverse cardiometabolic associations of adiposity begin earlier in the life course in males compared with females and are stronger until midlife. The findings may have implications for obesity prevention efforts, suggesting that adolescent and young adult males may be high priority targets for obesity prevention. Importantly, the findings also support the use of BMI as a general, easily measured and inexpensive method of adiposity measurement at a population level from childhood to mid adulthood. Future replication of these findings in independent cohorts using methods for causal inference is required to clarify if sex differences in associations found here reflect sex differences in causal effects. Future research examining sex-specific associations of adiposity with cardiometabolic traits in UK ethnic minorities is also required.

## Conclusions

The results of this study suggest that associations of adiposity with adverse cardiometabolic risk begin earlier in the life course in males compared with females and are stronger until midlife, particularly for key atherogenic lipids. Adolescent and young adult males may therefore be high priority targets for obesity prevention efforts.

## Supporting information

S1 Appendix Prespecified study protocol. (PDF)

S2 Appendix Further details on measurement of NMR traits. NMR, nuclear magnetic resonance.
(DOCX)

S3 Appendix STROBE checklist. STROBE, Strengthening the Reporting of Observational Studies in Epidemiology.
(DOCX)

S1 Table *P* values from LR tests of linearity testing at each time point. Numbers in columns here represent *P* values from LR tests examining linearity of sex-specific associations of measures of adiposity with outcomes at each age. *P* > 0.05 indicates no strong evidence of departure from linearity. BMI, body mass index; HDL, high-density lipoprotein; LDL, low-density lipoprotein; LR, likelihood ratio; VLDL, very-low-density lipoprotein.
(DOCX)

S2 Table Characteristics of offspring (G1 cohort) included in analyses compared to those excluded due to missing exposure, outcome or confounder data. ^a^Denominators for excluded participants in this table vary due to missing data for characteristics shown. ^b^Sample sizes vary due to missing data at each time point. BMI, body mass index; CSE, Certificate of Secondary Education; FM, fat mass; G1, offspring generation 1; SD, standard deviation; WC, waist circumference.
(DOCX)

S3 Table Characteristics of parents (G0 cohort) included in analyses compared to those excluded due to missing exposure, outcome or confounder data. ^a^Denominators for excluded participants in this table vary due to missing data for characteristics shown. ^b^Smoking during pregnancy is defined as the mother having self-reported smoking any type of tobacco in the first trimester. For males in G0, smoking during pregnancy is defined as the mother having reported on behalf of the father at 18 weeks gestation that the father currently smokes any type of tobacco. BMI, body mass index; CSE, Certificate of Secondary Education; FM, fat mass; G0, parent generation 0; SD, standard deviation; WC, waist circumference.
(DOCX)

S4 Table Adjusted sex-specific associations and sex difference in associations of BMI with 148 concentrations of cardiometabolic traits at multiple life stages. G1 analyses are adjusted for age at clinic completion, ethnicity, child’s mother and father education, maternal smoking during pregnancy, birth weight, gestational age, maternal age, household social class, and height and height^2^. Analyses of outcomes at 18 y and 25 y are also additionally adjusted for G1 offspring smoking. G0 analyses are adjusted for age at clinic completion, ethnicity, education, smoking during G1 cohort pregnancy, own social class, and height and height^2^. BMI, body mass index; G0, parent generation 0; G1, offspring generation 1; HDL, high-density lipoprotein; LCI, lower 95% confidence interval; LDL, low-density lipoprotein; UCI, upper 95% confidence interval; VLDL, very-low-density lipoprotein.
(XLSX)

S5 Table Adjusted sex-specific associations and sex differences in associations of fat mass with 148 concentrations of cardiometabolic traits at multiple life stages. G1 analyses are adjusted for age at clinic completion, ethnicity, child’s mother and father education, maternal smoking during pregnancy, birth weight, gestational age, maternal age, household social class, and height and height^2^. Analyses of outcomes at 18 y and 25 y are also additionally adjusted for G1 offspring smoking. G0 analyses are adjusted for age at clinic completion, ethnicity, education, smoking during G1 cohort pregnancy, own social class, and height and height^2^. G0, parent generation 0; G1, offspring generation 1; HDL, high-density lipoprotein; LCI, lower 95% confidence interval; LDL, low-density lipoprotein; UCI, upper 95% confidence interval; VLDL, very-low-density lipoprotein.
(XLSX)

S6 Table Adjusted sex-specific associations and sex differences in associations of waist circumference with 148 concentrations of cardiometabolic traits at multiple life stages. G1 analyses are adjusted for age at clinic completion, ethnicity, child’s mother and father education, maternal smoking during pregnancy, birth weight, gestational age, maternal age, household social class, and height and height^2^. Analyses of outcomes at 18 y and 25 y are also additionally adjusted for G1 offspring smoking. G0 analyses are adjusted for age at clinic completion, ethnicity, education, smoking during G1 cohort pregnancy, own social class, and height and height^2^. G0, parent generation 0; G1, offspring generation 1; HDL, high-density lipoprotein; LCI, lower 95% confidence interval; LDL, low-density lipoprotein; UCI, upper 95% confidence interval; VLDL, very-low-density lipoprotein; WC, waist circumference.
(XLSX)

S7 Table Adjusted sex-specific associations of BMI with concentrations of 148 cardiometabolic traits at multiple life stages—results in original units. G1 analyses are adjusted for age at clinic completion, ethnicity, child’s mother and father education, maternal smoking during pregnancy, birth weight, gestational age, maternal age, household social class, and height and height^2^. Analyses of outcomes at 18 y and 25 y are also additionally adjusted for G1 offspring smoking. G0 analyses are adjusted for age at clinic completion, ethnicity, education, smoking during G1 cohort pregnancy, own social class, and height and height^2^. BMI, body mass index; G0, parent generation 0; G1, offspring generation 1; HDL, high-density lipoprotein; LCI, lower 95% confidence interval; LDL, low-density lipoprotein; UCI, upper 95% confidence interval; VLDL, very-low-density lipoprotein.
(XLSX)

S8 Table Adjusted sex-specific associations of fat mass with concentrations of 148 cardiometabolic traits at multiple life stages—results in original units. G1 analyses are adjusted for age at clinic completion, ethnicity, child’s mother and father education, maternal smoking during pregnancy, birth weight, gestational age, maternal age, household social class, and height and height^2^. Analyses of outcomes at 18 y and 25 y are also additionally adjusted for G1 offspring smoking. G0 analyses are adjusted for age at clinic completion, ethnicity, education, smoking during G1 cohort pregnancy, own social class, and height and height^2^. G0, parent generation 0; G1, offspring generation 1; HDL, high-density lipoprotein; LCI, lower 95% confidence interval; LDL, low-density lipoprotein; UCI, upper 95% confidence interval; VLDL, very-low-density lipoprotein.
(XLSX)

S9 Table Adjusted sex-specific associations of waist circumference with concentrations of 148 cardiometabolic traits at multiple life stages—results in original units. G1 analyses are adjusted for age at clinic completion, ethnicity, child’s mother and father education, maternal smoking during pregnancy, birth weight, gestational age, maternal age, household social class, and height and height^2^. Analyses of outcomes at 18 y and 25 y are also additionally adjusted for G1 offspring smoking. G0 analyses are adjusted for age at clinic completion, ethnicity, education, smoking during G1 cohort pregnancy, own social class, and height and height^2^. G0, parent generation 0; G1, offspring generation 1; HDL, high-density lipoprotein; LCI, lower 95% confidence interval; LDL, low-density lipoprotein; UCI, upper 95% confidence interval; VLDL, very-low-density lipoprotein; WC, waist circumference.
(XLSX)

S10 Table Unadjusted sex-specific associations of BMI with concentrations of 148 cardiometabolic traits at multiple life stages. BMI, body mass index; G0, parent generation 0; G1, offspring generation 1; HDL, high-density lipoprotein; LCI, lower 95% confidence interval; LDL, low-density lipoprotein; UCI, upper 95% confidence interval; VLDL, very-low-density lipoprotein.
(XLSX)

S11 Table Unadjusted sex-specific associations of fat mass with concentrations of 148 cardiometabolic traits at multiple life stages. G0, parent generation 0; G1, offspring generation 1; HDL, high-density lipoprotein; LCI, lower 95% confidence interval; LDL, low-density lipoprotein; UCI, upper 95% confidence interval; VLDL, very-low-density lipoprotein.
(XLSX)

S12 Table Unadjusted sex-specific associations of waist circumference with concentrations of 148 cardiometabolic traits at multiple life stages. G0, parent generation 0; G1, offspring generation 1; HDL, high-density lipoprotein; LCI, lower 95% confidence interval; LDL, low-density lipoprotein; UCI, upper 95% confidence interval; VLDL, very-low-density lipoprotein; WC, waist circumference.
(XLSX)

S1 Fig Sex-specific association of BMI, fat mass, and waist circumference (per SD increase, standardised using sex-specific mean and SD) with standardised lipoprotein concentrations from childhood to midlife. Results shown are standardised differences with whiskers representing 95% CIs. These represent the standardised difference in cardiometabolic trait per SD increase in BMI, fat mass, and waist circumference in each sex separately for associations of adiposity at 9 y and traits at 15 y **(A)**, adiposity at 15 y and traits at 18 y **(B)**, adiposity at 18 y and traits at 25 y **(C)**, and adiposity at 50 y and traits at 50 y **(D)**. G1 analyses are adjusted for age at clinic completion, ethnicity, child’s mother and father education, maternal smoking during pregnancy, birth weight, gestational age, maternal age, household social class, and height and height^2^. Analyses of outcomes at 18 y and 25 y are also additionally adjusted for G1 offspring smoking. G0 analyses are adjusted for age at clinic completion, ethnicity, education, smoking during G1 cohort pregnancy, own social class, and height and height^2^. SD unit of BMI = 2.7 kg/m^2^, 2.9 kg/m^2^, 3.8 kg/m^2^, and 4.8 kg/m^2^ at 9 y, 15 y, 18 y, and 50 y, respectively. SD unit of fat mass = 5 kg, 7.8 kg, 9.8 kg, and 10.2 kg at 9 y, 15 y, 18 y, and 50 y, respectively. SD unit of waist circumference = 7.4 cm, 7.8 cm, and 13 cm at 9 y, 15 y, and 50 y, respectively. BMI, body mass index; CI, confidence interval; G0, parent generation 0; G1, offspring generation 1; HDL, high-density lipoprotein; LDL, low-density lipoprotein; SD, standard deviation; VLDL, very-low-density lipoprotein.
(DOCX)

S2 Fig Sex-specific association of BMI, fat mass, and waist circumference (per SD increase, standardised using sex-specific mean and SD) with standardised cholesterol, triglyceride, and other trait concentrations from childhood to midlife. Results shown are standardised differences with whiskers representing 95% CIs. These represent the standardised difference in cardiometabolic trait per SD increase in BMI, fat mass, and waist circumference in each sex separately for associations of adiposity at 9 y and traits at 15 y **(A)**, adiposity at 15 y and traits at 18 y **(B)**, adiposity at 18 y and traits at 25 y **(C)**, and adiposity at 50 y and traits at 50 y **(D)**. G1 analyses are adjusted for age at clinic completion, ethnicity, child’s mother and father education, maternal smoking during pregnancy, birth weight, gestational age, maternal age, household social class, and height and height^2^. Analyses of outcomes at 18 y and 25 y are also additionally adjusted for G1 offspring smoking. G0 analyses are adjusted for age at clinic completion, ethnicity, education, smoking during G1 cohort pregnancy, own social class, and height and height^2^. SD unit of BMI = 2.7 kg/m^2^, 2.9 kg/m^2^, 3.8 kg/m^2^, and 4.8 kg/m^2^ at 9 y, 15 y, 18 y, and 50 y, respectively. SD unit of fat mass = 5 kg, 7.8 kg, 9.8 kg, and 10.2 kg at 9 y, 15 y, 18 y, and 50 y, respectively. SD unit of waist circumference = 7.4 cm, 7.8 cm, and 13 cm at 9 y, 15 y, and 50 y, respectively, household social class, and height and height^2^. Analyses of outcomes at 18 y and 25 y are also additionally adjusted for G1 offspring smoking. G0 analyses are adjusted for age at clinic completion, ethnicity, education, smoking during G1 cohort pregnancy, own social class, and height and height^2^. BMI, body mass index; CI, confidence interval; G0, parent generation 0; G1, offspring generation 1; HDL, high-density lipoprotein; LDL, low-density lipoprotein; SD, standard deviation; VLDL, very-low-density lipoprotein.
(DOCX)

S3 Fig Sex-specific association of BMI, fat mass, and waist circumference (per SD increase) with standardised lipoprotein concentrations from childhood to midlife, including only complete family units. Results shown are standardised differences with whiskers representing 95% CIs. These represent the standardised difference in cardiometabolic trait per SD increase in BMI, fat mass, and waist circumference in each sex separately for associations of adiposity at 9 y and traits at 15 y **(A)**, adiposity at 15 y and traits at 18 y **(B)**, adiposity at 18 y and traits at 25 y **(C)**, and adiposity at 50 y and traits at 50 y **(D)**. G1 analyses are adjusted for age at clinic completion, ethnicity, child’s mother and father education, maternal smoking during pregnancy, birth weight, gestational age, maternal age, household social class, and height and height^2^. Analyses of outcomes at 18 y and 25 y are also additionally adjusted for G1 offspring smoking. G0 analyses are adjusted for age at clinic completion, ethnicity, education, smoking during G1 cohort pregnancy, own social class, and height and height^2^. SD unit of BMI = 2.7 kg/m^2^, 2.9 kg/m^2^, 3.8 kg/m^2^, and 4.8 kg/m^2^ at 9 y, 15 y, 18 y, and 50 y, respectively. SD unit of fat mass = 5 kg, 7.8 kg, 9.8 kg, and 10.2 kg at 9 y, 15 y, 18 y, and 50 y, respectively. SD unit of waist circumference = 7.4 cm, 7.8 cm, and 13 cm at 9 y, 15 y, and 50 y, respectively. BMI, body mass index; CI, confidence interval; G0, parent generation 0; G1, offspring generation 1; HDL, high-density lipoprotein; LDL, low-density lipoprotein; SD, standard deviation; VLDL, very-low-density lipoprotein.
(DOCX)

S4 Fig Sex-specific association of in BMI, fat mass, and waist circumference (per SD increase) with standardised cholesterol, triglyceride, and other trait concentrations from childhood to midlife, including only complete family units. Results shown are standardised differences with whiskers representing 95% CIs. These represent the standardised difference in cardiometabolic trait per SD increase in BMI, fat mass, and waist circumference in each sex separately for associations of adiposity at 9 y and traits at 15 y **(A)**, adiposity at 15 y and traits at 18 y **(B)**, adiposity at 18 y and traits at 25 y **(C)**, and adiposity at 50 y and traits at 50 y **(D)**. G1 analyses are adjusted for age at clinic completion, ethnicity, child’s mother and father education, maternal smoking during pregnancy, birth weight, gestational age, maternal age, household social class, and height and height^2^. Analyses of outcomes at 18 y and 25 y are also additionally adjusted for G1 offspring smoking. G0 analyses are adjusted for age at clinic completion, ethnicity, education, smoking during G1 cohort pregnancy, own social class, and height and height^2^. SD unit of BMI = 2.7 kg/m^2^, 2.9 kg/m^2^, 3.8 kg/m^2^, and 4.8 kg/m^2^ at 9 y, 15 y, 18 y, and 50 y, respectively. SD unit of fat mass = 5 kg, 7.8 kg, 9.8 kg, and 10.2 kg at 9 y, 15 y, 18 y, and 50 y, respectively. SD unit of waist circumference = 7.4 cm, 7.8 cm, and 13 cm at 9 y, 15 y, and 50 y, respectively. BMI, body mass index; CI, confidence interval; G0, parent generation 0; G1, offspring generation 1; HDL, high-density lipoprotein; LDL, low-density lipoprotein; SD, standard deviation; VLDL, very-low-density lipoprotein.
(DOCX)

S5 Fig Sex-specific association of BMI, fat mass, and waist circumference (per SD increase) with standardised lipoprotein concentrations from childhood to midlife, excluding participants in the top fifth of the adiposity distribution. Results shown are standardised differences with whiskers representing 95% CIs. These represent the standardised difference in cardiometabolic trait per SD increase in BMI, fat mass, and waist circumference in each sex separately for associations of adiposity at 9 y and traits at 15 y **(A)**, adiposity at 15 y and traits at 18 y **(B)**, adiposity at 18 y and traits at 25 y **(C)**, and adiposity at 50 y and traits at 50 y **(D)**. G1 analyses are adjusted for age at clinic completion, ethnicity, child’s mother and father education, maternal smoking during pregnancy, birth weight, gestational age, maternal age, household social class, and height and height^2^. Analyses of outcomes at 18 y and 25 y are also additionally adjusted for G1 offspring smoking. G0 analyses are adjusted for age at clinic completion, ethnicity, education, smoking during G1 cohort pregnancy, own social class, and height and height^2^. BMI, body mass index; CI, confidence interval; G0, parent generation 0; G1, offspring generation 1; HDL, high-density lipoprotein; LDL, low-density lipoprotein; SD, standard deviation; VLDL, very-low-density lipoprotein.
(DOCX)

S6 Fig Sex-specific association of in BMI, fat mass, and waist circumference (per SD increase) with standardised cholesterol, triglyceride, and other trait concentrations from childhood to midlife, excluding participants in the top fifth of the adiposity distribution. Results shown are standardised differences with whiskers representing 95% CIs. These represent the standardised difference in cardiometabolic trait per SD increase in BMI, fat mass, and waist circumference in each sex separately for associations of adiposity at 9 y and traits at 15 y **(A)**, adiposity at 15 y and traits at 18 y **(B)**, adiposity at 18 y and traits at 25 y **(C)**, and adiposity at 50 y and traits at 50 y **(D)**. G1 analyses are adjusted for age at clinic completion, ethnicity, child’s mother and father education, maternal smoking during pregnancy, birth weight, gestational age, maternal age, household social class, and height and height^2^. Analyses of outcomes at 18 y and 25 y are also additionally adjusted for G1 offspring smoking. G0 analyses are adjusted for age at clinic completion, ethnicity, education, smoking during G1 cohort pregnancy, own social class, and height and height^2^. BMI, body mass index; CI, confidence interval; G0, parent generation 0; G1, offspring generation 1; HDL, high-density lipoprotein; LDL, low-density lipoprotein; SD, standard deviation; VLDL, very-low-density lipoprotein.
(DOCX)

## Abbreviations

^1^H-NMR: proton nuclear magnetic resonance
A level: advanced level
ALSPAC: Avon Longitudinal Study of Parents and Children
BMI: body mass index
CHD: coronary heart disease
CI: confidence interval
CSE: certificate of secondary education
CVD: cardiovascular disease
DAG: directed acyclic graph
DXA: dual-energy X-ray absorptiometry
FM: fat mass
G0: Generation 0
G1: Generation 1
HDL: high-density lipoprotein
LDL: low-density lipoprotein
LR: likelihood ratio
O level: ordinary level
SBP: systolic blood pressure
SD: standard deviation
SOC: standard occupational classification
STROBE: Strengthening the Reporting of Observational Studies in Epidemiology
T2DM: type 2 diabetes mellitus
VLDL: very-low-density lipoprotein
WC: waist circumference
