# Correction: Sex-specific associations of adiposity with cardiometabolic traits in the UK: A multi-life stage cohort study with repeat metabolomics

**DOI:** 10.1371/journal.pmed.1004901

**Published:** 2026-01-23

**Authors:** Linda M. O’Keeffe, Joshua A. Bell, Kate N. O’Neill, Matthew A. Lee, Mark Woodward, Sanne A. E. Peters, George Davey Smith, Patricia M. Kearney

In the Funding statement, the grant number from the funder The Wellcome Trust is listed incorrectly. The correct grant number is: 092731/Z/10/Z.
