# Supplementary material for: Sex-specific associations of adiposity with cardiometabolic traits in the UK: A multi–life stage cohort study with repeat metabolomics
Source: PLoS Med. 2022 Jan 6;19(1):e1003636. doi: 10.1371/journal.pmed.1003636 (PMC8735621; doi:10.1371/journal.pmed.1003636)
Supplement: S1 Appendix — (PDF) [file pmed.1003636.s001.pdf]

Submission information

SCaRLeT: Sex differences in Cardiovascular Risk across Life course Transitions (B3199)

Submission date/time: 24/10/2018 12:56:45

Principal Applicant Details

Principal Applicant Title: Dr

Your name: Linda O'Keeffe

Position Held (note: If you are a student, the proposal should be submitted by your supervisor with you listed as a co-applicant): Research Fellow

Is this a student project?: No

What type of student project is this?:

Please specify what type of student project this is...:

Affiliation: University College Cork

Are you a 'direct access' user of ALSPAC data, or a member of the Integrative Epidemiology Unit (IEU)?: No

Address line 1: College Road

Address line 2:

City: Cork

Country: Ireland

Postcode: T12 K8AF

Email (Please use your institutional email address): linda.okeeffe@ucc.ie

Telephone: +353 (0)21 490 3000

Co-applicant details

How many co-applicants?: 6

Co-applicant 1

Name and title: Prof Patricia Kearney
Position Held and Affiliation: Professor of Epidemiology, University College Cork
Email: patricia.kearney@ucc.ie
Role in Project: Mentor

Co-applicant 2

Name and title: Dr Tony Fitzgerald
Position Held and Affiliation: Senior Lecturer in Biostatistics, University College Cork
Email: t.fitzgerald@ucc.ie
Role in Project: Co-investigator

Co-applicant 3

Name and title: Dr Darren Dahly
Position Held and Affiliation: Senior Lecturer in Research Methods
Email: ddahly@ucc.ie
Role in Project: Co-investigator

Co-applicant 4

Name and title: Professor George Davey-Smith
Position Held and Affiliation: Professor of Clinical Epidemiology, University of Bristol
Email: kz.davey-smith@bristol.ac.uk
Role in Project: Co-investigator

Co-applicant 5

Name and title: Professor Kate Tilling
Position Held and Affiliation: Professor of Medical Statistics
Email: kate.tilling@bristol.ac.uk
Role in Project: Co-investigator

Co-applicant 6

Name and title: Professor Cecilia Lindgren

Position held and affiliation: Professor of Genomic Endocrinology and Metabolism
Email: celi@broadinstitute.org
Role in project: Co-investigator

Co-applicant 7

Co-applicant 8

Co-applicant 9

Co-applicant 10

PROJECT DETAILS

Title of project: SCaRLeT: Sex differences in Cardiovascular Risk across Life course Transitions

Proposed Project Start Date: 2019-09-01

Proposed Project Finish Date: 2023-09-01

Is your project currently funded?: No

Current project funding:

Are you seeking funding for this project?: Yes

----------------Potential funding fields----------------
- **Potential project funding:** Health Research Board of Ireland Emerging Investigator Award

- **Funder's deadline:** 2018-12-17

Will your project be a cross-cohort project or part of a consortium?: No

Please provide a description of how ALSPAC data will be combined with other datasets, how it will be managed and how many researchers are likely to access it.:

Have you previously had a project with us?: Yes

Data buddy name (if one has been assigned to you):

Where did you find out about ALSPAC?: Academic paper

Please specify where you heard about ALSPAC?:

Research dissemination: Peer review journal article, Conference, Other, please describe

Other plans for disseminating research findings: Where applicable, findings of this work, as appropriate may also be disseminated via policy briefs.

----------------Invoice details----------------
- **Name:** Linda O'Keeffe

- **Position Held:** Research Fellow

- **Address:** Oakfield House,

Oakfield Grove,

Bristol,

UK

- **Email:** linda.okeeffe@bristol.ac.uk

- **Telephone number:** 07534498919

Name of legal signatory for your institution (e.g. representative from contracts department):

Link to the information security policy for your institution:

Description of Project

Project summary for laypersons: Cardiovascular disease (CVD) is the leading cause of death worldwide and personalised prevention of well-established modifiable CVD risk factors such as smoking and obesity are a global public health priority. Striking sex differences in CVD risk exist through adult life. Modifiable CVD risk factors such as smoking begin in childhood and track through the life course. In adults, there is evidence of sex-specific associations of modifiable risk factors and CVD risk. However, this evidence has many limitations including: limited abilities to make causal inferences about sex-specific cardiovascular risk due to biases in observational studies; a lack of focus on when and how modifiable risk factors influence sex-specific cardiovascular risk (a life course approach), and; limited understanding of the mediators of sex-specific cardiovascular risk (the steps linking cause and effect).

Aim(s) and objective(s): Using life course epidemiology and sophisticated

methods that improve causal inference, this project aims to improve understanding of sex-specific modifiable risk factors for CVD across the life course. It will i) examine sex-specific associations of modifiable risk factors and cardiovascular risk using different methods for causal inference to unpick the role of bias in evidence from observational studies; ii) identify when and how modifiable risk factors begin to affect cardiovascular risk in a sex-specific manner, which may provide insights about when to target prevention and; iii) identify mediators of sex-specific effects to provide insight into underlying mechanisms and pathways.

Methods (including an overview of statistical methods): Life course models will be the primary methods used in the analysis of this data; these will include multilevel models,structural equation models or a structured life course approach, as appropriate, to examine the association between sex-specific life course trajectories of modifiable risk factors and measures of cardiovascular risk.

Exposures, outcomes and confounders to be considered (justifying particular types of data as necessary): Key exposures in ALSPAC offspring: DEXA measures of fat and lean mass from age 9; measures of BMI from birth; measures of smoking,alcohol use and physical activity across the entire follow-up period from late childhood.

Key mediators/outcomes in ALSPAC offspring: measures of blood pressure from age 7 onward; measures of insulin and glucose at all available clinics to the latest follow-up clinic; measures of lipids from birth onward; measures of c-reactive protein and other emerging risk factors across the follow-up period; measures of pulse wave velocity, carotid intima media thickness and left ventricular mass index at ages 18 and 25 years.

Key confounders/covariates in ALSPAC offspring: measures of maternal smoking, education and other maternal variables as a measure of child socio-economic position; measures of reproductive function in females and males such as age at peak height velocity and age at menarche.

Reasons for using ALSPAC: ALSPAC is a unique resource which can address the research question of interest here in a way that no other resource can, given the repeated measurement of risk factors for cardiovascular risk from birth, through childhood and adolescence and into early adulthood.

What do you think the likely impact of your research will be?: This research will increase our understanding of the sex-specific aetiology of cardiovascular risk across the early life course and may potentially inform sex-specific prevention opportunities downstream.

Subject classification (please select one): Epidemiology

Other subject - please specify:

----------------Diseases/conditions (click to expand/collapse)----------------
- **Please tick all appropriate diseases/conditions:** Other (please specify)

- **Other disease/condition - please specify:** Cardiovascular disease risk

----------------Techniques (click to expand/collapse)----------------
- **Please tick all appropriate techniques:** Statistical methods

- **Other techniques - please specify:**

----------------Keywords (click to expand/collapse)----------------
- **Please tick all appropriate keywords:** Cardiovascular

- **Methods - please specify:**

- **Other keyword(s) (please specify):**

Types of existing data required

Does your project involve analysing existing data?: Yes
Existing questionnaire data: Yes

Existing clinic data: Yes

Existing data from biological samples (not genetic): Yes

----------------Existing genome-wide SNP genotype data----------------
- **Existing genetics data:** Yes

The following genetics data options were submitted before changes were made to the genetic dataset labels in May 2021.
The submitted values have been shown based on the current dataset labels.

Dataset labels in the proposal form have changed in the following way:

Dataset group: Genome-wide array genotype data
Previous label | Current label
Mother | Genome-wide - Illumina 660 quad - G0 mothers
Father | Genome-wide - Illumina exome core array - G0 partners
Child | Genome-wide - Illumina 550 quad - G1 child

Dataset group: Genome-wide array based imputation - HapMap2
Previous label | Current label
Mother | Genome-wide - HapMap2 imputed - G0 mothers
Child | Genome-wide - HapMap2 imputed - G1 child

Dataset group: Genome-wide array based imputation - 1000 genomes
Previous label | Current label
Mothers and children | Genome-wide - 1000G imputed - G0 mothers + G1 child

Fathers | Genome-wide - 1000G imputed - G0 partners

Dataset group: Genome-wide array based imputation - HRC
Previous label | Current label
Mother | [No longer provided]
Child | [No longer provided]
[new combined dataset] | Genome-wide - HRC imputed - G0 mothers + G1 child

Dataset group: Genome-wide array based imputation - HLA (new group)
Previous label | Current label
[new dataset] | HLA imputation - G1 child

Dataset group: Genome-wide array based copy number variants
Previous label | Current label
Child | Genome-wide - CNV - G1 child

Dataset group: Genome sequencing
Previous label | Current label
UK 10K | Whole genome sequencing - G1 child
[new dataset] | Whole exome sequencing - G1 child

Dataset group: Gene expression
Previous label | Current label
Child | Gene expression - array - G1 child

- **Genome-wide array genotype data:** Genome-wide - Illumina 550 quad - G1 child

- **Genome-wide array based imputation - HapMap2:** Genome-wide - HapMap2 imputed - G1 child

- **Genome-wide array based imputation - 1000 genomes:** Genome-wide - 1000G imputed - G0 mothers + G1 child

- **Genome-wide array based imputation - HRC:**

- **Genome-wide array based imputation - HLA:**

- **Genome-wide array based copy number variants:**

- **Genome sequencing:**

- **Gene expression:**

- **Do you have additional genetics data requirements?:**

- **Additional genetics data requirements:**

The following genetics data options are now retired but were specified as part of this proposal submission:

- **Genome-wide array based imputation - HRC:**

- **Specific genotypes:** No

- **SNPs:**

- **SNPs list:**

- **Variants within the following range:**

- **Other variants (e.g. VNTRs):**

- **Other variants list:**

----------------Existing Methylation Data----------------
- **Methylation data:** No

----------------Linkage data----------------
- **Third party data (e.g. data provided by education/health organisations):** No

----------------Address Data----------------
- **Do you require any data linked to addresses?:** No

----------------Text data----------------
- **Text data:** No

NEW data or sample collection

Are you requesting the collection of new data and/or biosamples?: No
----------------New data/biosamples----------------
- **Please name the person responsible for handling any incidental findings from the data / sample collection:**

- **Please provide details of what data you want to collect:**

- **How will your data be collected?:**

Processing of samples

----------------Do you want new genotyping carried out by LGC?----------------
- **SNP genotyping:** No

----------------Do you want new genotyping or methylation data generated on the Illumina platform?----------------
- **Do you require Illumina arrays to be run in the ALSPAC Laboratory?:** No

----------------Do you require DNA samples for other analysis?----------------
- **Do you require DNA samples to analyse elsewhere?:** No

Non-DNA biological samples

Will the project require access to biological samples other than DNA?: No

----------------Other biological samples----------------

Terms and Conditions

1. Please confirm that...: ...you are familiar with the latest version of the ALSPAC access policy (http://www.bristol.ac.uk/alspac/researchers/access)

2. Please confirm that...: ...you understand it is your responsibility to ensure that all members of your team working on this project complete a Data User Responsibilities Agreement (DURA) and that you inform ALSPAC of any changes to the team

3. Please confirm that...: ...you understand that data and samples from the ALSPAC resource cannot be used for commercial gain

4. Please confirm that...: ...you understand that you and your team must not pass on any data or samples awarded, or any derived variables or genotypes generated by this application to a third party (i.e. to anybody who is not included in the list of applicants on this form)

5. Please confirm that...: ...you aware that any third party seeking to use data, samples, or derived variables or genotypes arising from this application must approach ALSPAC to obtain access permission of their own?

6. Please confirm that...: ...you understand that if a problem arises involving any misuse of the ALSPAC data or samples provided for this project that violates any of the terms and conditions specified by the Data Access Agreement (DAA) that you (as principal applicant) have signed, you will be held responsible. This might result in you being excluded from using the ALSPAC resource in the future.

7. I understand that...: ...costs will be determined after the proposal has been approved and that I will not receive any data or samples until I have settled my invoice or provided a purchase order number.

8. I understand that...: ...all genotypes and/or data generated from biological samples will be returned to ALSPAC and be made available to other researchers.

9. I declare that...: ...I have no conflict of interest in relation to this research.

If you do have a conflict on interest, please declare it here:

Date: 2018-10-24

Print name (this will serve as your signature): LINDA O KEEFFE
