# Supplementary material for: Sex-specific associations of adiposity with cardiometabolic traits in the UK: A multi–life stage cohort study with repeat metabolomics
Source: PLoS Med. 2022 Jan 6;19(1):e1003636. doi: 10.1371/journal.pmed.1003636 (PMC8735621; doi:10.1371/journal.pmed.1003636)
Supplement: S2 Appendix — NMR, nuclear magnetic resonance. (DOCX) [file pmed.1003636.s002.docx]

**S2 Appendix Further details on measurement of NMR traits**

For each sample, the NMR spectra were analysed for absolute metabolite quantification (molar concentration) in automated fashion. A ridge regression model was applied for quantification of each metabolite to overcome the problems of heavily overlapping spectral data. Quantification of lipoprotein lipid data was performed by calibrating against high performance liquid chromatography methods, and then individually cross-validated against NMR-independent lipid data. Low-molecular-weight metabolites, as well as lipid extract measures, were quantified as mmol/l based on regression modelling calibrated against a set of manually fitted metabolite measures. The calibration data was quantified based on iterative line-shape fitting analysis using PERCH NMR software (PERCH Solutions Ltd., Kuopio, Finland). Absolute quantification could not be directly established for the lipid extract measures due to experimental variation in the lipid extraction protocol. Therefore, serum extract metabolites have been scaled via the total cholesterol as quantified from the native serum LIPO spectrum. We have previously shown strong correlation between the NMR and clinical chemistry measures that are available from both methods [1, 2]. Further detailed information on the platform including details for sample preparation, NMR analysis, and data processing/quantitation are described in detail elsewhere [3-5].

1. O'Keeffe L, Simpkin A, Tilling K, Anderson E, Hughes A, Lawlor DA, et al. Sex-specific trajectories of cardiometabolic risk factors during childhood and adolescence: a prospective cohort study. Atherosclerosis. 2018;278:190-6.

2. O'Keeffe L, Simpkin A, Tilling K, Anderson E, Hughes A, Lawlor DA, et al. Data on trajectories of measures of cardiovascular health in the Avon Longitudinal Study of Parents and Children (ALSPAC). Data Brief. 2019;23(103687).

3. Soininen P, Kangas AJ, Würtz P, Tukiainen T, Tynkkynen T, Laatikainen R, et al. High-throughput serum NMR metabonomics for cost-effective holistic studies on systemic metabolism. Analyst. 2009;134(9):1781-5.

4. Inouye M, Kettunen J, Soininen P, Silander K, Ripatti S, Kumpula LS, et al. Metabonomic, transcriptomic, and genomic variation of a population cohort. Mol Syst Biol. 2010;6(1):441.

5. Kettunen J, Tukiainen T, Sarin A-P, Ortega-Alonso A, Tikkanen E, Lyytikäinen L-P, et al. Genome-wide association study identifies multiple loci influencing human serum metabolite levels. Nat Genet. 2012;44(3):269-76.
