# Supplementary material for: Sex-specific associations of adiposity with cardiometabolic traits in the UK: A multi–life stage cohort study with repeat metabolomics
Source: PLoS Med. 2022 Jan 6;19(1):e1003636. doi: 10.1371/journal.pmed.1003636 (PMC8735621; doi:10.1371/journal.pmed.1003636)
Supplement: S1 Table — Numbers in columns here represent P values from LR tests examining linearity of sex-specific associations of measures of adiposity with outcomes at each age. P > 0.05 indicates no strong evidence of departure from linearity. BMI, body mass index; HDL, high-density lipoprotein; LDL, low-density lipoprotein; LR, likelihood ratio; VLDL, very-low-density lipoprotein. (DOCX) [file pmed.1003636.s004.docx]

**S1 Table P values from likelihood ratio tests of linearity testing at each time point**

|  | **BMI** | | **Waist Circumference** | | **Fat Mass** | |
| --- | --- | --- | --- | --- | --- | --- |
|  | **Females** | **Males** | **Females** | **Males** | **Females** | **Males** |
| **Adiposity measure at age 9 and metabolic trait at age 15** | | | | | | |
| Total lipids in chylomicrons and extremely large VLDL (mmol/l) | 0.114 | 0.136 | 0.197 | 0.290 | 0.173 | 0.481 |
| Total lipids in very large VLDL (mmol/l) | 0.160 | 0.117 | 0.193 | 0.166 | 0.287 | 0.480 |
| Total lipids in large VLDL (mmol/l) | 0.230 | 0.115 | 0.217 | 0.110 | 0.388 | 0.565 |
| Total lipids in medium VLDL (mmol/l) | 0.257 | 0.133 | 0.258 | 0.126 | 0.338 | 0.545 |
| Total lipids in small VLDL (mmol/l) | 0.162 | 0.345 | 0.397 | 0.421 | 0.174 | 0.566 |
| Total lipids in very small VLDL (mmol/l) | 0.553 | 0.765 | 0.955 | 0.683 | 0.235 | 0.674 |
| Total lipids in large LDL (mmol/l) | 0.405 | 0.088 | 0.546 | 0.151 | 0.461 | 0.432 |
| Total lipids in medium LDL (mmol/l) | 0.338 | 0.068 | 0.618 | 0.206 | 0.419 | 0.512 |
| Total lipids in small LDL (mmol/l) | 0.323 | 0.066 | 0.664 | 0.218 | 0.413 | 0.495 |
| Total lipids in very large HDL (mmol/l) | 0.182 | 0.023 | 0.055 | 0.145 | 0.413 | 0.003 |
| Total lipids in large HDL (mmol/l) | 0.148 | 0.029 | 0.018 | 0.077 | 0.263 | 0.004 |
| Total lipids in medium HDL (mmol/l) | 0.669 | 0.287 | 0.352 | 0.179 | 0.367 | 0.206 |
| Total lipids in small HDL (mmol/l) | 0.453 | 0.448 | 0.735 | 0.100 | 0.519 | 0.829 |
| Apolipoprotein A-I (g/l) | 0.326 | 0.017 | 0.102 | 0.131 | 0.459 | 0.006 |
| Apolipoprotein B (g/l) | 0.322 | 0.444 | 0.909 | 0.991 | 0.190 | 0.830 |
| Serum total cholesterol (mmol/l) | 0.459 | 0.104 | 0.472 | 0.168 | 0.540 | 0.215 |
| Free cholesterol (mmol/l) | 0.544 | 0.138 | 0.523 | 0.334 | 0.339 | 0.263 |
| Esterified cholesterol (mmol/l) | 0.435 | 0.101 | 0.410 | 0.108 | 0.650 | 0.208 |
| Remnant cholesterol (non-HDL, non-LDL -cholesterol) (mmol/l) | 0.565 | 0.705 | 0.983 | 0.896 | 0.218 | 0.717 |
| Total cholesterol in VLDL (mmol/l) | 0.357 | 0.327 | 0.671 | 0.627 | 0.140 | 0.557 |
| Total cholesterol in LDL (mmol/l) | 0.416 | 0.108 | 0.589 | 0.131 | 0.470 | 0.493 |
| Total cholesterol in HDL (mmol/l) | 0.271 | 0.021 | 0.066 | 0.102 | 0.311 | 0.003 |
| Serum total triglycerides (mmol/l) | 0.133 | 0.224 | 0.305 | 0.221 | 0.280 | 0.633 |
| Triglycerides in VLDL (mmol/l) | 0.188 | 0.150 | 0.194 | 0.117 | 0.354 | 0.531 |
| Triglycerides in LDL (mmol/l) | 0.208 | 0.002 | 0.326 | 0.796 | 0.457 | 0.245 |
| Triglycerides in HDL (mmol/l) | 0.496 | 0.456 | 0.811 | 0.415 | 0.465 | 0.735 |
|  |  |  |  |  |  |  |
| **Adiposity measure at age 15 and metabolic trait at age 18** | | | | | | |
| Total lipids in chylomicrons and extremely large VLDL (mmol/l) | 0.001 | 0.155 | 0.298 | 0.726 | 0.124 | 0.444 |
| Total lipids in very large VLDL (mmol/l) | 0.002 | 0.163 | 0.432 | 0.690 | 0.087 | 0.382 |
| Total lipids in large VLDL (mmol/l) | 0.003 | 0.214 | 0.500 | 0.674 | 0.063 | 0.374 |
| Total lipids in medium VLDL (mmol/l) | 0.002 | 0.407 | 0.392 | 0.715 | 0.051 | 0.467 |
| Total lipids in small VLDL (mmol/l) | 0.004 | 0.405 | 0.337 | 0.898 | 0.148 | 0.391 |
| Total lipids in very small VLDL (mmol/l) | 0.007 | 0.231 | 0.090 | 0.387 | 0.299 | 0.173 |
| Total lipids in large LDL (mmol/l) | 0.120 | 0.450 | 0.360 | 0.291 | 0.332 | 0.187 |
| Total lipids in medium LDL (mmol/l) | 0.107 | 0.503 | 0.378 | 0.394 | 0.328 | 0.204 |
| Total lipids in small LDL (mmol/l) | 0.083 | 0.565 | 0.361 | 0.435 | 0.325 | 0.192 |
| Total lipids in very large HDL (mmol/l) | 0.347 | 0.804 | 0.030 | 0.543 | 0.517 | 0.287 |
| Total lipids in large HDL (mmol/l) | 0.040 | 0.973 | 0.002 | 0.384 | 0.426 | 0.601 |
| Total lipids in medium HDL (mmol/l) | 0.040 | 0.969 | 0.014 | 0.400 | 0.865 | 0.851 |
| Total lipids in small HDL (mmol/l) | 0.019 | 0.689 | 0.104 | 0.514 | 0.989 | 0.451 |
| Apolipoprotein A-I (g/l) | 0.064 | 0.755 | 0.015 | 0.225 | 0.505 | 0.817 |
| Apolipoprotein B (g/l) | 0.009 | 0.444 | 0.266 | 0.737 | 0.114 | 0.449 |
| Serum total cholesterol (mmol/l) | 0.084 | 0.541 | 0.418 | 0.209 | 0.300 | 0.199 |
| Free cholesterol (mmol/l) | 0.153 | 0.419 | 0.565 | 0.254 | 0.191 | 0.120 |
| Esterified cholesterol (mmol/l) | 0.071 | 0.624 | 0.394 | 0.157 | 0.408 | 0.248 |
| Remnant cholesterol (non-HDL, non-LDL -cholesterol) (mmol/l) | 0.006 | 0.414 | 0.162 | 0.542 | 0.149 | 0.360 |
| Total cholesterol in VLDL (mmol/l) | 0.001 | 0.498 | 0.132 | 0.839 | 0.110 | 0.559 |
| Total cholesterol in LDL (mmol/l) | 0.150 | 0.509 | 0.341 | 0.297 | 0.304 | 0.172 |
| Total cholesterol in HDL (mmol/l) | 0.029 | 0.872 | 0.003 | 0.319 | 0.421 | 0.807 |
| Serum total triglycerides (mmol/l) | 0.009 | 0.135 | 0.836 | 0.735 | 0.109 | 0.404 |
| Triglycerides in VLDL (mmol/l) | 0.005 | 0.214 | 0.518 | 0.674 | 0.074 | 0.351 |
| Triglycerides in LDL (mmol/l) | 0.019 | 0.033 | 0.200 | 0.895 | 0.759 | 0.541 |
| Triglycerides in HDL (mmol/l) | 0.044 | 0.269 | 0.619 | 0.797 | 0.267 | 0.913 |
|  |  |  |  |  |  |  |
| **Adiposity measure at age 18 and metabolic trait at age 25** | | | | | | |
| Total lipids in chylomicrons and extremely large VLDL (mmol/l) | 0.170 | 0.567 | 0.158 | 0.251 | - | - |
| Total lipids in very large VLDL (mmol/l) | 0.134 | 0.614 | 0.123 | 0.317 | - | - |
| Total lipids in large VLDL (mmol/l) | 0.082 | 0.615 | 0.132 | 0.293 | - | - |
| Total lipids in medium VLDL (mmol/l) | 0.062 | 0.528 | 0.131 | 0.315 | - | - |
| Total lipids in small VLDL (mmol/l) | 0.105 | 0.302 | 0.313 | 0.321 | - | - |
| Total lipids in very small VLDL (mmol/l) | 0.555 | 0.297 | 0.999 | 0.584 | - | - |
| Total lipids in large LDL (mmol/l) | 0.722 | 0.805 | 0.772 | 0.919 | - | - |
| Total lipids in medium LDL (mmol/l) | 0.871 | 0.687 | 0.724 | 0.868 | - | - |
| Total lipids in small LDL (mmol/l) | 0.921 | 0.642 | 0.570 | 0.823 | - | - |
| Total lipids in very large HDL (mmol/l) | 0.227 | 0.057 | 0.002 | 0.382 | - | - |
| Total lipids in large HDL (mmol/l) | 0.145 | 0.135 | 0.010 | 0.308 | - | - |
| Total lipids in medium HDL (mmol/l) | 0.241 | 0.392 | 0.231 | 0.104 | - | - |
| Total lipids in small HDL (mmol/l) | 0.593 | 0.088 | 0.070 | 0.192 | - | - |
| Apolipoprotein A-I (g/l) | 0.269 | 0.624 | 0.110 | 0.250 | - | - |
| Apolipoprotein B (g/l) | 0.738 | 0.543 | 0.980 | 0.704 | - | - |
| Serum total cholesterol (mmol/l) | 0.757 | 0.916 | 0.465 | 0.778 | - | - |
| Free cholesterol (mmol/l) | 0.940 | 0.856 | 0.666 | 0.850 | - | - |
| Esterified cholesterol (mmol/l) | 0.751 | 0.936 | 0.469 | 0.747 | - | - |
| Remnant cholesterol (non-HDL, non-LDL -cholesterol) (mmol/l) | 0.663 | 0.466 | 0.981 | 0.641 | - | - |
| Total cholesterol in VLDL (mmol/l) | 0.276 | 0.320 | 0.644 | 0.308 | - | - |
| Total cholesterol in LDL (mmol/l) | 0.787 | 0.764 | 0.761 | 0.897 | - | - |
| Total cholesterol in HDL (mmol/l) | 0.119 | 0.318 | 0.051 | 0.151 | - | - |
| Serum total triglycerides (mmol/l) | 0.124 | 0.538 | 0.274 | 0.333 | - | - |
| Triglycerides in VLDL (mmol/l) | 0.058 | 0.556 | 0.115 | 0.320 | - | - |
| Triglycerides in LDL (mmol/l) | 0.930 | 0.600 | 0.729 | 0.514 | - | - |
| Triglycerides in HDL (mmol/l) | 0.856 | 0.691 | 0.192 | 0.249 | - | - |
|  |  |  |  |  |  |  |
| **Adiposity measure at age 50 and metabolic trait at age 50** | | | | | | |
| Total lipids in chylomicrons and extremely large VLDL (mmol/l) | 0.00004 | 0.417 | 0.0003 | 0.361 | 0.177 | 0.799 |
| Total lipids in very large VLDL (mmol/l) | 0.00002 | 0.577 | 0.0002 | 0.308 | 0.111 | 0.808 |
| Total lipids in large VLDL (mmol/l) | 0.00002 | 0.562 | 0.0003 | 0.058 | 0.282 | 0.528 |
| Total lipids in medium VLDL (mmol/l) | 0.00008 | 0.681 | 0.004 | 0.009 | 0.740 | 0.592 |
| Total lipids in small VLDL (mmol/l) | 0.007 | 0.413 | 0.098 | 0.0002 | 0.554 | 0.659 |
| Total lipids in very small VLDL (mmol/l) | 0.616 | 0.026 | 0.787 | 0.0001 | 0.006 | 0.005 |
| Total lipids in large LDL (mmol/l) | 0.960 | 0.006 | 0.472 | 0.003 | 0.040 | 0.001 |
| Total lipids in medium LDL (mmol/l) | 0.987 | 0.016 | 0.414 | 0.006 | 0.062 | 0.002 |
| Total lipids in small LDL (mmol/l) | 0.984 | 0.020 | 0.379 | 0.007 | 0.049 | 0.002 |
| Total lipids in very large HDL (mmol/l) | 0.387 | 0.348 | 0.046 | 0.0002 | 0.123 | 0.917 |
| Total lipids in large HDL (mmol/l) | 0.056 | 0.497 | 0.064 | 0.001 | 0.166 | 0.654 |
| Total lipids in medium HDL (mmol/l) | 0.294 | 0.489 | 0.382 | 0.910 | 0.406 | 0.209 |
| Total lipids in small HDL (mmol/l) | 0.799 | 0.311 | 0.042 | 0.349 | 0.575 | 0.325 |
| Apolipoprotein A-I (g/l) | 0.369 | 0.377 | 0.015 | 0.887 | 0.386 | 0.076 |
| Apolipoprotein B (g/l) | 0.398 | 0.124 | 0.254 | 0.0001 | 0.143 | 0.073 |
| Serum total cholesterol (mmol/l) | 0.976 | 0.017 | 0.263 | 0.017 | 0.080 | 0.001 |
| Free cholesterol (mmol/l) | 0.988 | 0.041 | 0.249 | 0.031 | 0.246 | 0.003 |
| Esterified cholesterol (mmol/l) | 0.948 | 0.013 | 0.140 | 0.020 | 0.041 | 0.001 |
| Remnant cholesterol (non-HDL, non-LDL -cholesterol) (mmol/l) | 0.292 | 0.137 | 0.380 | 0.0002 | 0.090 | 0.049 |
| Total cholesterol in VLDL (mmol/l) | 0.009 | 0.470 | 0.121 | 0.0003 | 0.394 | 0.338 |
| Total cholesterol in LDL (mmol/l) | 0.957 | 0.005 | 0.442 | 0.003 | 0.027 | 0.001 |
| Total cholesterol in HDL (mmol/l) | 0.046 | 0.500 | 0.085 | 0.131 | 0.282 | 0.153 |
| Serum total triglycerides (mmol/l) | 0.000 | 0.710 | 0.002 | 0.010 | 0.530 | 0.614 |
| Triglycerides in VLDL (mmol/l) | 0.000 | 0.652 | 0.002 | 0.015 | 0.539 | 0.529 |
| Triglycerides in LDL (mmol/l) | 0.772 | 0.151 | 0.128 | 0.009 | 0.682 | 0.068 |
| Triglycerides in HDL (mmol/l) | 0.024 | 0.742 | 0.001 | 0.060 | 0.185 | 0.502 |

**Legend:** BMI, body mass index**;** HDL, high-density lipoprotein cholesterol; LDL, low-density lipoprotein cholesterol; VLDL, very-low-density lipoprotein cholesterol. Numbers in columns here represent P values from likelihood ratio tests examining linearity of sex-specific associations of measures of adiposity with outcomes at each age. P >0.05 indicates no strong evidence of departure from linearity.
