# Supplementary material for: Sex-specific associations of adiposity with cardiometabolic traits in the UK: A multi–life stage cohort study with repeat metabolomics
Source: PLoS Med. 2022 Jan 6;19(1):e1003636. doi: 10.1371/journal.pmed.1003636 (PMC8735621; doi:10.1371/journal.pmed.1003636)
Supplement: S2 Table — aDenominators for excluded participants in this table vary due to missing data for characteristics shown. bSample sizes vary due to missing data at each time point. BMI, body mass index; CSE, Certificate of Secondary Education; FM, fat mass; G1, offspring generation 1; SD, standard deviation; WC, waist circumference. (DOCX) [file pmed.1003636.s005.docx]

**S2 Table Characteristics of offspring (G1 cohort) included in analyses compared to those excluded due to missing exposure, outcome or confounder data**

|  | **Female participants included**  **n= 1,629** | **Female participants excluded**  **n=4,108-5,077 ^a^** | **Male participants included**  **n= 1,452** | **Male participants excluded**  **n=4,705-5,856^a^** |
| --- | --- | --- | --- | --- |
|  | **n (%)** | **n (%)** | **n (%)** | **n (%)** |
| **UK ethnic minorities** | 25 (1.5) | 127 (2.9) | 25 (1.7) | 147 (3.0) |
| **Maternal Education** |  |  |  |  |
| CSE | 152 (9.3) | 1047 (23.8) | 111 (7.6) | 1210 (24.3) |
| Vocational | 104 (6.4) | 483 (11.0) | 96 (6.6) | 545 (10.9) |
| O level | 551 (32.8) | 1535 (34.9) | 478 (32.9) | 1753 (35.2) |
| A level | 479 (29.4) | 882 (20.1) | 465 (32.0) | 968 (19.4) |
| Degree | 343 (21.1) | 451 (10.3) | 302 (20.8) | 510 (10.2) |
| **Fathers’ education** |  |  |  |  |
| CSE | 256 (15.7) | 1263 (30.2) | 182 (12.5) | 1428 (30.3) |
| Vocational | 113 (6.9) | 389 (9.3) | 109 (7.5) | 402 (8.5) |
| O level | 337 (20.7) | 881 (21.1) | 327 (22.5) | 1006 (21.3) |
| A level | 497 (30.5) | 1044 (25.0) | 416 (28.7) | 1159 (24.6) |
| Degree | 426 (26.2) | 608 (14.5) | 418 (28.8) | 723 (15.3) |
|  |  |  |  |  |
| **Smoking in pregnancy** | 225 (13.8) | 1326 (27.7) | 186 (12.8) | 1597 (29.6) |
| **Household social class** |  |  |  |  |
| Professional | 304 (18.6) | 426 (10.7) | 315 (21.7) | 492 (11.0) |
| Managerial & Technical | 772 (47.4) | 1584 (39.8) | 717 (49.4) | 1749 (39.0) |
| Non-Manual | 404 (24.8) | 1026 (25.8) | 315 (21.7) | 1200 (26.7) |
| Manual | 111 (6.8) | 643 (16.1) | 82 (5.7) | 725 (16.2) |
| Part Skilled & Unskilled | 38 (2.3) | 306 (7.7) | 23 (1.6) | 324 (7.2) |
|  | **Mean (SD)** | ***Mean (SD)*** | ***Mean (SD)*** | ***Mean (SD)*** |
| ***Age 9 ^b^*** |  |  |  |  |
| **BMI (kg/m^2^)** | 17.7 (2.8) | 18.1 (3.2) | 17.3 (2.5) | 17.6 (2.9) |
| **Fat mass (kg)** | 9.4 (4.8) | 10.0 (5.3) | 7.2 (4.6) | 7.5 (5.0) |
| **WC (cm)** | 62.1 (7.3) | 63.0 (8.4) | 62.8 (7.1) | 63.4 (8.0) |
| ***Age 15 ^b^*** |  |  |  |  |
| **BMI (kg/m^2^)** | 21.7 (3.5) | 22.0 (3.9) | 20.9 (3.1) | 21.2 (3.6) |
| **Fat mass (kg)** | 18.7 (8.0) | 19.3 (8.8) | 11.0 (8.0) | 11.8 (8.9) |
| **WC at (cm)** | 76.7 (8.6) | 76.9 (9.5) | 76.1 (8.4) | 77.2 (9.5) |
| ***Age 18 ^b^*** |  |  |  |  |
| **BMI (kg/m^2^)** | 22.7 (4.0) | 23.4 (4.9) | 22.4 (3.7) | 23.0 (4.1) |
| **Fat mass (kg)** | 21.1 (8.9) | 22.4 (10.4 | 13.4 (9.6) | 14.8 (10.4)) |
|  |  |  |  |  |
| **Birthweight (g)** | 3.39 (0.48) | 3.31 (0.56) | 3.48 (0.57) | 3.42 (0.62) |
| **Gestational age (weeks)** | 39.6 (1.6) | 39.3 (2.6) | 39.4 (1.8) | 39.1 (2.7) |
| **Maternal age (years)** | 29.5 (4.3) | 27.8 (4.8) | 29.7 (4.3) | 28.0 (5.0) |

Legend: BMI, body mass index; CSE, Certificate of Secondary Education; FM, fat mass; G1, generation 1; SD, standard deviation; WC, waist circumference. ^a^ Denominators for excluded participants in this table vary due to missing data for characteristics shown. ^b^ Sample sizes vary due to missing data at each time point.
