# Supplementary material for: Sex-specific associations of adiposity with cardiometabolic traits in the UK: A multi–life stage cohort study with repeat metabolomics
Source: PLoS Med. 2022 Jan 6;19(1):e1003636. doi: 10.1371/journal.pmed.1003636 (PMC8735621; doi:10.1371/journal.pmed.1003636)
Supplement: S3 Table — aDenominators for excluded participants in this table vary due to missing data for characteristics shown. bSmoking during pregnancy is defined as the mother having self-reported smoking any type of tobacco in the first trimester. For males in G0, smoking during pregnancy is defined as the mother having reported on behalf of the father at 18 weeks gestation that the father currently smokes any type of tobacco. BMI, body mass index; CSE, Certificate of Secondary Education; FM, fat mass; G0, parent generation 0; SD, standard deviation; WC, waist circumference. (DOCX) [file pmed.1003636.s006.docx]

**S3 Table Characteristics of parents (G0 cohort) included in analyses compared to those excluded due to missing exposure, outcome or confounder data**

|  | **Female participants included**  **n=3,446** | **Female participants excluded**  **n=6,540-9,730 ^a^** | **Male participants included**  **n=1,441** | **Male participants excluded**  **n=9,449-10,767 ^a^** |
| --- | --- | --- | --- | --- |
|  | **n (%)** | **n (%)** | **n (%)** | **n (%)** |
| **UK ethnic minorities** | 64 (1.9) | 257 (2.9) | 17 (1.2) | 459 (4.4) |
| **Education** |  |  |  |  |
| CSE | 273 (7.9) | 2220 (25.0) | 136 (9.4) | 2961 (28.5) |
| Vocational | 233 (6.8) | 982 (11.1) | 78 (5.4) | 923 (8.9) |
| O level | 1188 (34.5) | 3079 (34.7) | 300 (20.8) | 2216 (21.3) |
| A level | 1064 (30.9) | 1700 (19.2) | 420 (29.2) | 2662 (25.6) |
| Degree | 688 (20.0) | 898 (10.1) | 507 (35.2) | 1642 (15.8) |
| **Smoking in pregnancy ^b^** | 457 (13.3) | 2856 (29.4) | 253 (17.6) | 4113 (38.2) |
| **Household social class** |  |  |  |  |
| Professional | 280 (8.1) | 310 (4.7) | 278 (19.3) | 915 (9.7) |
| Managerial & Technical | 1318 (38.3) | 1820 (27.8) | 619 (43.0) | 3076 (32.6) |
| Non-Manual | 1378 (40.0) | 2891 (44.2) | 170 (11.8) | 1015 (10.7) |
| Manual | 202 (5.9) | 580 (8.9) | 275 (19.1) | 3142 (33.3) |
| Part Skilled & Unskilled | 268 (7.8) | 939 (14.4) | 99 (6.9) | 1301 (13.8) |
|  | ***Mean (SD)*** | ***Mean (SD)*** | ***Mean (SD)*** | ***Mean (SD)*** |
| **BMI (kg/m^2^)** | 26.4 (5.1) | 28.2 (4.5) | 27.4 (3.9) | 27.3 (6.0) |
| **Fat mass (kg)** | 26.7 (10.6) | 24.8 (9.8) | 23.4 (8.9) | 28.4 (11.6) |
| **WC (cm)** | 84.0 (12.0) | 99.0 (11.9) | 97.2 (10.5) | 86.4 (13.6) |

Legend: BMI, body mass index; CSE, Certificate of Secondary Education; FM, fat mass; G0, generation 0; SD, standard deviation; WC, waist circumference. ^a^ Denominators for excluded participants in this table vary due to missing data for characteristics shown. ^b^ Smoking during pregnancy is defined as the mother having self-reported smoking any type of tobacco in the first trimester. For males in G0, smoking during pregnancy is defined as the mother having reported on behalf of the father (during the first trimester) that the father currently smokes any type of tobacco.
