# Supplementary material for: Sex-specific associations of adiposity with cardiometabolic traits in the UK: A multi–life stage cohort study with repeat metabolomics
Source: PLoS Med. 2022 Jan 6;19(1):e1003636. doi: 10.1371/journal.pmed.1003636 (PMC8735621; doi:10.1371/journal.pmed.1003636)
Supplement: S2 Fig — Results shown are standardised differences with whiskers representing 95% CIs. These represent the standardised difference in cardiometabolic trait per SD increase in BMI, fat mass, and waist circumference in each sex separately for associations of adiposity at 9 y and traits at 15 y (A), adiposity at 15 y and traits at 18 y (B), adiposity at 18 y and traits at 25 y (C), and adiposity at 50 y and traits at 50 y (D). G1 analyses are adjusted for age at clinic completion, ethnicity, child’s mother and father education, maternal smoking during pregnancy, birth weight, gestational age, maternal age, household social class, and height and height2. Analyses of outcomes at 18 y and 25 y are also additionally adjusted for G1 offspring smoking. G0 analyses are adjusted for age at clinic completion, ethnicity, education, smoking during G1 cohort pregnancy, own social class, and height and height2. SD unit of BMI = 2.7 kg/m2, 2.9 kg/m2, 3.8 kg/m2, and 4.8 kg/m2 at 9 y, 15 y, 18 y, and 50 y, respectively. SD unit of fat mass = 5 kg, 7.8 kg, 9.8 kg, and 10.2 kg at 9 y, 15 y, 18 y, and 50 y, respectively. SD unit of waist circumference = 7.4 cm, 7.8 cm, and 13 cm at 9 y, 15 y, and 50 y, respectively, household social class, and height and height2. Analyses of outcomes at 18 y and 25 y are also additionally adjusted for G1 offspring smoking. G0 analyses are adjusted for age at clinic completion, ethnicity, education, smoking during G1 cohort pregnancy, own social class, and height and height2. BMI, body mass index; CI, confidence interval; G0, parent generation 0; G1, offspring generation 1; HDL, high-density lipoprotein; LDL, low-density lipoprotein; SD, standard deviation; VLDL, very-low-density lipoprotein. (DOCX) [file pmed.1003636.s017.docx]

**S2 Figure Sex-specific association of BMI, fat mass and waist circumference (per SD increase, standardised using sex-specific mean and SD) with standardised cholesterol, triglyceride and other trait concentrations from childhood to midlife. Legend:** BMI, body mass index; G1, offspring generation 1; G0, parent generation 0; HDL, high-density lipoprotein; LDL, low-density lipoprotein; VLDL, very-low-density lipoprotein. Results shown are standardised differences with whiskers representing 95% confidence intervals. These represent the standardised difference in cardiometabolic trait per standard deviation increase in BMI, fat mass and waist circumference in each sex separately for associations of adiposity at 9y and traits at 15y (A), adiposity at 15y and traits at 18y (B), adiposity at 18y and traits at 25y (C) and adiposity at 50y and traits at 50y (D). G1 analyses are adjusted for age at clinic completion, ethnicity, child’s mother and father education, [maternal](https://www.sciencedirect.com/topics/medicine-and-dentistry/gravidity-and-parity) smoking during pregnancy, birthweight, gestational age, maternal age, household social class and height and height^2^. Analyses of outcomes at 18y and 25y are also additionally adjusted for G1 offspring smoking. G0 analyses are adjusted for age at clinic completion, ethnicity, education, smoking during G1 cohort pregnancy, own social class and height and height^2^.SD unit of BMI = 2.7 kg/m^2^, 2.9 kg/m^2^, 3.8 kg/m^2^ and 4.8 kg/m^2^ at 9y, 15y, 18y and 50y respectively. SD unit of fat mass = 5kg, 7.8 kg, 9.8kg and 10.2kg at 9y, 15y, 18y and 50y respectively. SD unit of waist circumference = 7.4cm, 7.8cm and 13cm at 9y, 15y and 50y respectively., household social class and height and height^2^. Analyses of outcomes at 18y and 25y are also additionally adjusted for G1 offspring smoking. G0 analyses are adjusted for age at clinic completion, ethnicity, education, smoking during G1 cohort pregnancy, own social class and height and height^2^.
